# PedMap: a pediatric diseases map generated from clinical big data from Hangzhou, China

**DOI:** 10.1038/s41598-019-54439-w

**Published:** 2019-11-28

**Authors:** Haomin Li, Gang Yu, Cong Dong, Zheng Jia, Jiye An, Huilong Duan, Qiang Shu

**Affiliations:** 1 The Children’s Hospital of Zhejiang University School of Medicine and National Clinical Research Center for Child Health, Hangzhou, China; 2 College of Biomedical Engineering and Instrument Science, Zhejiang University, Hangzhou, China

**Keywords:** Paediatric research, Information technology

## Abstract

Epidemiological knowledge of pediatric diseases may improve professionals’ understanding of the pathophysiology of and risk factors for diseases and is also crucial for decision making related to workforce and resource planning in pediatric departments. In this study, a pediatric disease epidemiology knowledgebase called PedMap (http://pedmap.nbscn.org) was constructed from the clinical data from 5 447 202 outpatient visits of 2 189 868 unique patients at a children’s hospital (Hangzhou, China) from 2013 to 2016. The top 100 most-reported pediatric diseases were identified and visualized. These common pediatric diseases were clustered into 4 age groups and 4 seasons. The prevalence, age distribution and co-occurrence diseases for each disease were also visualized. Furthermore, an online prediction tool based on Gaussian regression models was developed to predict pediatric disease incidence based on weather information. PedMap is the first comprehensive epidemiological resource to show the full view of age-related, seasonal, climate-related variations in and co-occurrence patterns of pediatric diseases.

## Introduction

Many countries experience significant shortages in pediatric specialists^1–3^. China has the largest population of children in the world. According to a report from UNICEF (the United Nations International Children’s Emergency Fund), approximately 271 million children between 0 and 17 years were in China in 2015^4^. At the same time, there are only 118 000 registered pediatricians in China^5^. The shortage of pediatricians and specialized children’s hospitals in China is a well-known challenge^2,6^. With such a pediatric resource shortage, comprehensive epidemiological knowledge of pediatric diseases may improve professionals’ understanding of the pathophysiology and risk factors of disease and is also crucial for decision making related to workforce and resource planning in pediatric departments. The goal of this study is to identify the most common pediatric diseases and their age distributions, seasonal variations and co-occurrence patterns.

Age plays an important role in pediatric medicine and is also an important factor when considering phenotypic changes in health and diseases states^7^. Some researchers have described age-related patterns of diseases^8^. However, there is no report on how to classify common pediatric diseases into age groups thus far. The recognition of the seasonality of disease occurrence has been longstanding, dating at least to the time of Hippocrates (∼380 BC). Seasonal variations in pediatric diseases have has been well described in many infectious diseases (e.g., HFMD^9^, pneumonia and gastroenteritis^10^), several chronic diseases (e.g., asthma^11^ and diabetes^12^) and other diseases (e.g., dermatoses^13^ and epistaxis^14^). However, the seasonality of diseases is not always consistent around the world, given the diverse meteorological environment and developing/developed statuses^15^; some studies have even shown conflicting results and age-related differentiation^14^. Seasons are divisions of the year marked by weather changes. Therefore, the seasonality of diseases is a comprehensive reflection of correlations among various meteorological features and diseases. Local long-term meteorological data and large cohort studies always have more accuracy in revealing these patterns than other types of studies. Several studies on co-occurrence diseases focus on depression^16^, diseases of immune dysfunction^17^ and chronic diseases among older adults^18^. Recently, an eHDN (epidemiological human disease network) was published to show the interconnections among human diseases^19^. However, a network that shows the relationships among pediatric diseases is still not available.

Traditional epidemiological studies often require long-term data collection efforts. The accumulated clinical practice big data in recent years provides an opportunity to glean new actionable knowledge, especially knowledge concerning children’s health^20,21^. To explore the age distribution and seasonality and co-occurrence patterns in pediatrics, we analyzed all first outpatient visits (n = 5 447 202) to our children’s hospital over a 4-year period (2013–2016). Based on data mining, clustering and association analysis with meteorological data, an epidemiological knowledgebase of pediatric diseases called PedMap was constructed and published at http://pedmap.nbscn.org.

## Methods

### Pediatric disease data

This retrospective study was approved by the Institutional Review Board/Ethics Committee of the Children’s Hospital of Zhejiang University School of Medicine (Hangzhou, China). All research was performed in accordance with relevant guidelines and regulations. Written informed consent was waived by the Institutional Review Board/Ethics Committee, as the utilization of anonymized retrospective data does not require patient consent under the local legislation. According to a government statistical report, at the end of 2018, there were about 12.2 million children (<14 years old) in Hangzhou, the capital and most populous city of Zhejiang Province in East China with an area of 34,585 km^2^. Given its status as the center of children’s healthcare in Zhejiang Province and the National Clinical Research Center for Child Health in China, the number of daily outpatient visits in this hospital often exceeds 10 000 visits. Because patients do not require a referral to visit children’s hospitals as outpatients in China, the disease spectrum of outpatients is an adequate reflection of the disease spectrum of the population of children in this area. We collected the diagnosis of all patients who first visited our children’s hospital for a certain condition between January 1, 2013, and December 31, 2016. During this period, there were 5 447 202 outpatient visits involving 2 189 868 unique patients with 6 433 different diagnosis terms. We selected the 100 most common pediatric diseases based on the monthly average incidence of diseases. Then, we manually coded the 100 most common pediatric diseases with ICD-10 (the 10^th^ revision of the International Statistical Classification of Diseases and Related Health Problems) for further analysis. As physicians use different levels of diagnosis granularity in practice, some codes can be very similar, such as those for diarrhea (K52.916) and acute enteritis (K52.904), but most diagnoses have with only 4-length codes, such as pneumonia (J18.9). If two diagnoses are coded with the same ICD-10 code, the incidence data of the two diagnoses will be combined. The ages in months when the patients visited were also collected for each outpatient visit. Then, the means, standard deviations and median ages for the 100 most common pediatric diseases were calculated. For each disease, the histograms for age in intervals of 1 month were also generated by normalizing the distribution with the maximum value. The disease incidences for each disease and each month was normalized as a z-scores, which is the signed fractional numbers of standard deviations by which the incidence is above or below the overall mean across the 48 months.

### Age and seasonal pattern recognition

Both the normalized age histograms over 216 months (18 years) and incidence z-scores over 48 months are type of longitudinal data. We used the kml package in R (version 3.4.0) to cluster the longitudinal data based on k-means clustering^22^, as it is not possible to know beforehand the optimum number of clusters. The kml package provides a graphical interface for choosing the ‘best’ number of clusters. The age group and seasonal knowledge were also used to choose the best number of patterns. More details about the data preprocessing procedure and clustering of the longitudinal data are described in the supplemental methods section.

### Climate data and regression model

We collected the following weather data for each day in Hangzhou during the study period from the Weather Underground website (www.wunderground.com): temperature (°C) [average, high and low], dew point (°C) [average, high and low], sea level pressure (hPa) [average, high and low], humidity (%) [average, high, low], visibility (km) [average], wind speed (km/h) [average, high and gust wind] and amount of rainfall (mm). The monthly averages of these climate features were calculated and combined with the normalized incidence of each disease to support the Pearson correlation analysis and Gaussian regression analysis using the cor() and glm() R functions, respectively. Because there is always a time lag between weather changes and patient visits, we also created weekly climate features and disease incidence datasets to perform a cross-correlation analysis and identify the lag relationships among them^23^.

### Co-occurrence odds ratios and co-occurrence network

Among the top 100 common pediatric diseases, the odds ratio (OR) of one disease cooccurring with another disease, which means that the same patient visited the center for both of these two diseases during the study period, but not necessarity in the same visit, were calculated by performing Fisher’s exact test in R (version 3.4.0). Diseases with a log(OR) greater than 2 were used to construct a pediatric disease co-occurrence network.

### Other methods used in this study

The distances between pairs of ICD-10 codes of the 100 most common pediatric diseases were calculated to build a distance matrix (details about this calculation are described in the supplemental methods section). Then, this distance matrix was mapped to 2D space using multidimensional scaling (*cmdscale* in R version 3.4.0)^24^.

## Results

### Top 100 most common pediatric diseases

Based on the average incidence per month during the four years of the study, the top 100 most common pediatric diseases were selected. These diseases accounted for more than 78% of all the outpatient visits. As shown in Supplemental Figure S1, the top 100 diseases were plotted by size depending on the average incidence per month and were colored by disease categories in the ICD-10 coding system. The most enhanced ICD-10 category is J (diseases of the respiratory system), and acute upper respiratory infection is the top disease, occurring approximately 20 240 times per month on average. In total, 19 diseases of the respiratory system, such as bronchitis, pneumonia and acute pharyngitis, are included in the top 100 most common pediatric diseases. The next most detailed ICD-10 category is R (symptoms, signs and abnormal clinical and laboratory findings), which includes the most common symptoms in children, such as fever, abdominal pain, cough and vomiting. The third popular category is K (diseases of the digestive system), such as dyspepsia and enteritis. In addition, the top 100 most common pediatric diseases covered most of the ICD-10 categories, such as L30 (dermatitis), N47 (diseases of the genitourinary system, especially phimosis), F90–98 (behavioral and emotional disorders with onset usually occurring in childhood and adolescence, especially developmental disorders and hyperkinetic disorders), E (endocrine, nutritional and metabolic diseases, such as hypothyroidism and disorders of puberty), H (diseases of the eye and ear, such as conjunctivitis, ametropia and otitis media), B (viral infections, especially herpangina), Q (congenital malformations and deformations, such as ankyloglossia and concealed penises), G (diseases of the nervous system, such as epilepsy), P07 (preterm infants), S09 (injuries of the head), D18 (hemangiomas) and Z01 (dental examinations). However, the distribution of incidence among the diseases is not balanced: the top 10 diseases account for more than 41% of all the outpatient visits and more than half of the outpatient visits for the top 100 diseases. A summary of the seasonal and age patterns of common pediatric diseases is shown in Supplemental Table S1 and is explained in the following sections.

### The age distribution of the top 100 most common pediatric diseases

The age distribution of all outpatient visits is shown in Supplemental Figure S2. Overall, there is a downward trend with increasing age, but there are two peaks at approximately 6 months and 40 months. The means and standard deviations of age for the top 100 pediatric diseases were calculated and shown in Supplemental Figure S3. Several diseases affect only small infants, such as P59.9 (neonatal jaundice), J21.9 (pediatric bronchiolitis) and H04.4 (chronic dacryocystitis). There are also several diseases that affect only older children, especially diseases from ICD-10 category E (endocrine, nutritional and metabolic diseases), such as E66.9 (obesity) and E34.3 (short stature). The mean age for most diseases ranges from 20 to 80 months, and the mean age for high-incidence pediatric diseases is approximately 3 years old. The standard deviation ranged from 4.556 to 64.242 months and showed very different age distributions for different diseases. The standard deviation is positively correlated with the mean age of onset for the disease (R = 0.485), meaning that some of the diseases affect only certain children at younger ages.

### The age patterns for common pediatric diseases

The normalized age distributions of the top 100 diseases are shown in Supplemental Figure S4. At first glance, there are different types of distributions. We used the kml package to cluster these 100 age distributions using a k value ranging from 2 to 10 and reran the model 5 times with different initial conditions. The theoretically “best” k value for clustering was 2 for imbalanced distributions, as shown in Supplemental Figure S5. However, we chose to use k = 4 for clustering because it could provide more detailed clustering results and relatively good clustering quality (based on the Calinski & Harabatz index^25^).

**Figure 1:**
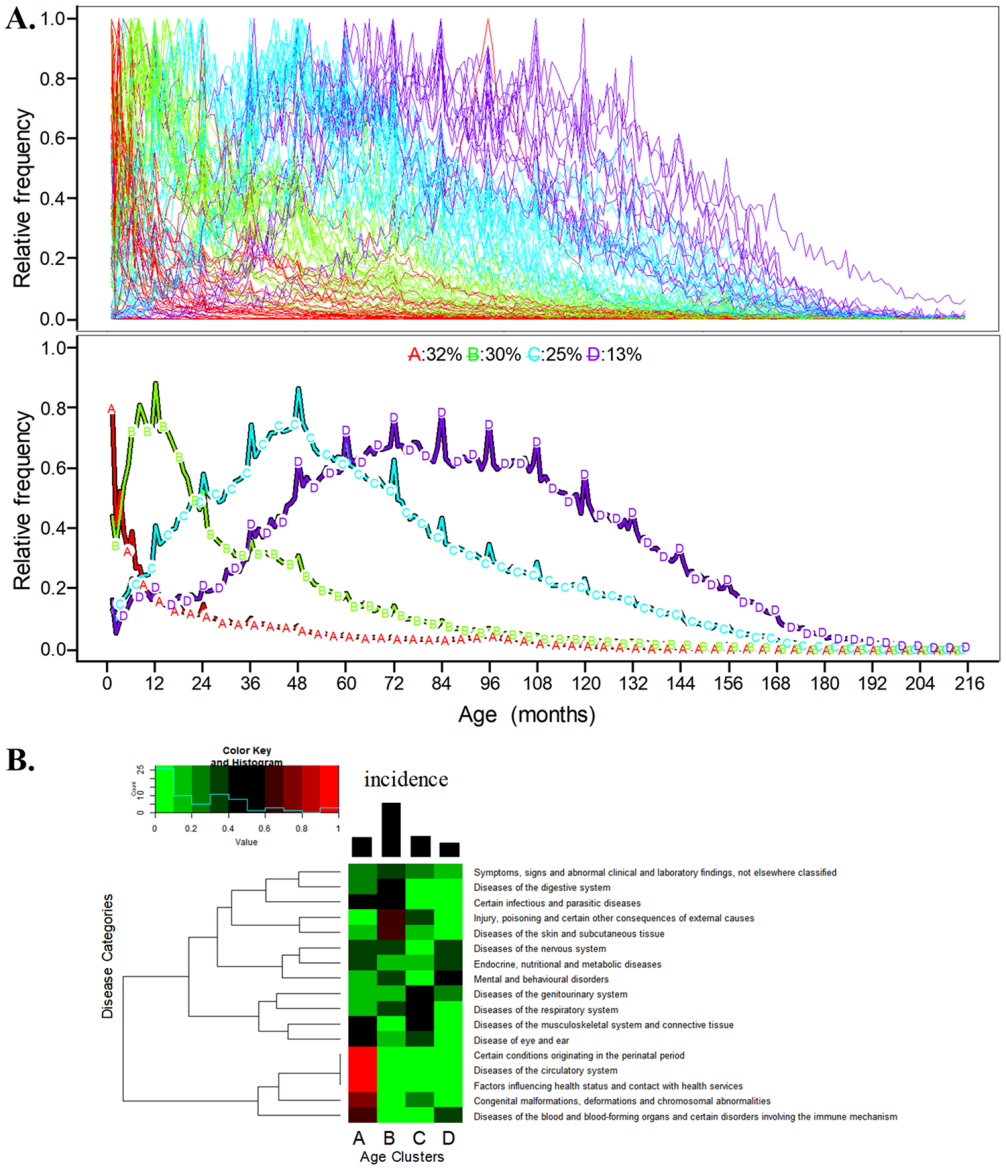
(**A**) The clustering results of the age distributions for 100 common pediatric diseases. The top section shows the original age distribution trajectories of the 100 diseases. The lower section shows the trajectories of the means of the 4 clusters, which are based on k-means clustering for longitudinal data: Cluster A (young infants), Cluster B (infant), Cluster C (preschool children) and Cluster D (schoolchildren). (**B**) The disease distribution in the 4 age clusters. The heatmap shows the percentage of different disease categories distributed in the 4 age clusters as shown in (**A**). The bar chart on the top shows the incidence for these age clusters.

As shown in Fig. 1A, Cluster A contains 32 diseases that mostly affect young infants, and Cluster B contains 30 diseases that mainly affect infants who are approximately 1 year old. The diseases in Clusters C and D, which have wider peaks, primarily affect the preschool children and schoolchildren, respectively. There are noticeable spikes of the mean trajectories because some of the birthdate registered to the system do not have month granularity and are set to the year.

The distribution of different disease categories in the 4 age clusters is shown in Fig. 1B. Cluster A (young infants) is characterized by conditions originating in the perinatal period, congenital malformations and diseases of the circulation system. Cluster B (infants) includes injuries, skin diseases, and digestive system diseases. Cluster C (preschool children) was mainly affected by genitourinary and respiratory diseases. Cluster D (schoolchildren) was primarily affected by mental and behavioral disorders and endocrine, nutritional and metabolic diseases. The incidence of disease in Cluster B (infants) was the highest among the 4 clusters.

### The season features of 100 common pediatric diseases

**Figure 2:**
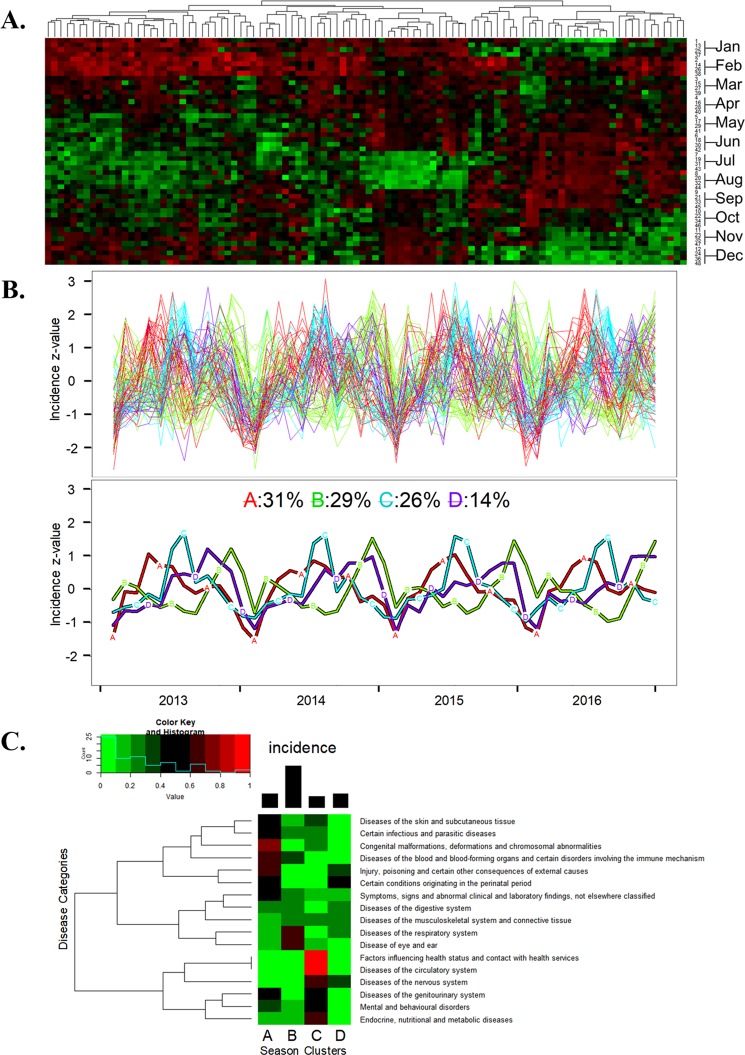
Seasonality of the top 100 pediatric diseases. (**A**) The heatmap of the normalized incidence of the 100 most common pediatric diseases across 4 years in different months. (High incidence z-scores is green, and low incidence z-score are red). (**B**) The clustering results of the seasonal pattern of the 100 most common pediatric diseases. The top section shows the original disease incidence trajectories of the 100 diseases over 48 months. The lower section shows the trajectories of the means of the 4 clusters based on k-means clustering for longitudinal data: Cluster A (spring), Cluster B (winter), Cluster C (summer), Cluster D (autumn). (**C**) The heatmap shows the percentage of different disease categories distributed in season clusters as shown in (**B**). The bar chart on the top shows the incidence for these season clusters.

As shown in Fig. 2A, the seasonal variation in pediatric diseases is clear, and some of the diseases are well known to pediatricians in practice. To identify the four seasons associated with pediatric diseases, k = 4 was also used to cluster the 100 common pediatric diseases, as shown in Fig. 2B. Cluster A, which contains 31 diseases, shows a spring peak, and Cluster C, which contains 26 diseases, shows a summer peak. Only 14 diseases included in Cluster D were common in autumn. The most common diseases in the winter were classified into Cluster B. The distribution of different disease categories in the 4 seasonal patterns is shown in Fig. 2C. This figure shows that the most common viral infections, such as tonsillitis, pharyngitis and herpangina, occurred in the spring (Cluster A), and diseases of the circulatory, nervous, and genitourinary systems and endocrine, nutritional and metabolic diseases are common in the summer (Cluster C). In the autumn (Cluster D), diseases of the digestive system, such as dyspepsia, diarrhea and abdominal pain, were frequent. Diseases in the winter (Cluster B), which have a high average incidence, included diseases of the respiratory system, such as AURIs, bronchitis, fever and pneumonia.

### The associations among disease incidence and weather features

**Figure 3:**
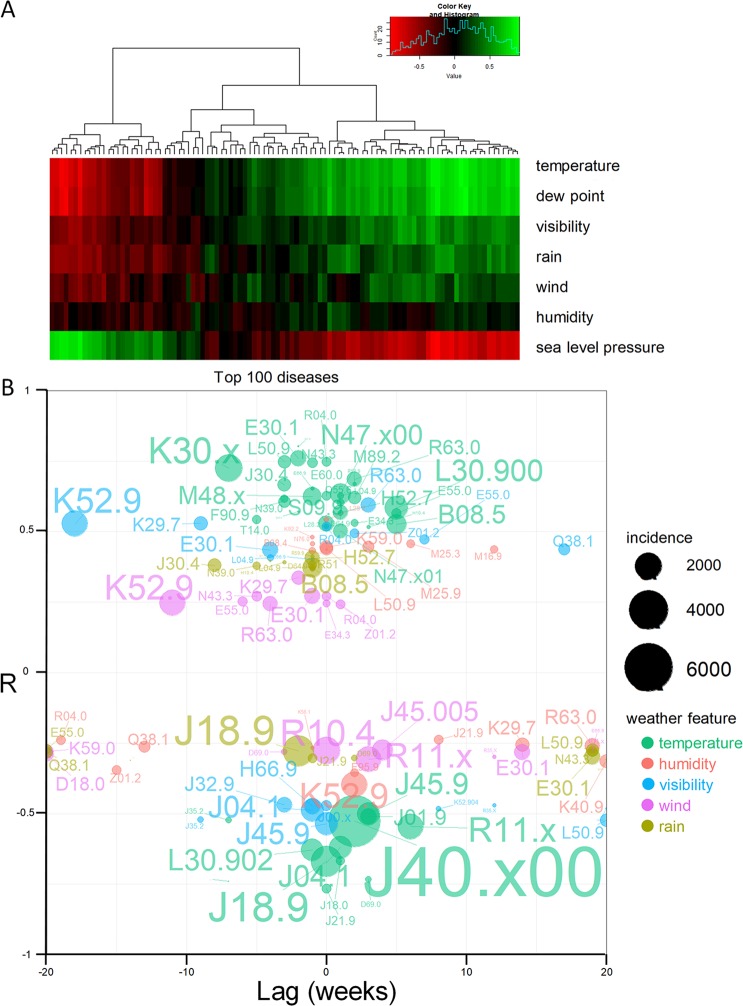
Correlation of weather features with disease incidence. (**A**) The heatmap of the correlation between weather features and the incidence of the 100 most common pediatric diseases. Red and green indicate negative and positive correlation, respectively, and the black indicates the no correlations. (**B**) The cross-correlation analysis identified the correlation coefficients and time lags between weather features and disease incidence.

Seasons are divisions of the year marked by changes in the weather, ecology, and the amount of daylight. Therefore, the seasonality of pediatric diseases is a comprehensive reflection of correlations among various meteorological features and diseases. The univariate correlations among monthly weather features and disease incidence were calculated and plotted as a heatmap in Fig. 3A.

Many diseases were highly correlated with weather features (the light red and green represent the strong negative and positive correlation, the detail of the correlation coefficient and corresponding p-value were shown in Supplemental Table S2), especially temperature and temperature-related features (dew point and sea-level pressure). The diseases of the respiratory systems were mainly clustered on the left side of the heatmap, which indicates a negative correlation with temperature and humidity. The amount of precipitation and visibility in the summer are always higher than those in the other seasons. Therefore, they also show correlation patterns similar to those related to temperature. The correlations among humidity, wind and disease incidence were relatively weak, and only a few diseases, such as papular urticaria and eczema, are correlated with high humidity and low humidity, respectively.

The temporal relationships among different weather features and the incidence of common pediatric diseases were analyzed based on cross-correlation analysis across ±20 weeks. The cross-correlation absolute R values larger than 0.5 or the top 10 correlated diseases of a weather feature are shown in Fig. 3B. As the dew point and sea-level pressure are highly correlated with temperature, diseases correlated with these characteristics were ignored in Fig. 3B. Diseases of the respiratory system, such as pediatric bronchiolitis (J21.9), pneumonia (J18.9), bronchitis (J40.x00) and asthmatic bronchitis (J45.9), showed a negative correlation with temperature, and the incidence peaked slightly before the temperature valley (the lowest point of temperature). The peak for vomiting (R11.x) usually occurred 6 weeks before the temperature decreased to the valley. The diseases that were positively correlated with temperature are more widely distributed and diverse, including epistaxis (R04.0), thrush (B37.9), hydroceles (N43.3) and some summer-vacation-related patient visits, such as those for precocity (E30.1), obesity (E66.9), phimosis (N47.x00) and growth retardation (M89.2). Some injuries were also correlated with temperature due to less protection by clothes and more outdoor activities in the summer, such as skin traumas (T14.0), fractures (M48.x) and head injuries (S09.9). Dyspepsia (K30.x) usually occurs 7 weeks after the temperature peak. Skin diseases, such as dermatitis (L30.900), eczema (L30.902) and urticaria (L50.9), are weather-sensitive but have different features: Dermatitis (L30.900) is positively correlated with temperature at lag 5, but eczema (L30.902) is negatively correlated with temperature at lag −1. Urticaria (L50.9) was positively correlated with humidity but negatively correlated with visibility and wind speed. Correlations among pediatric diseases and other weather features, such as humidity, visibility, wind speed and amount of rainfall, were relatively weak. Only some correlations exceeded 0.5, such as those for asthmatic bronchitis (J45.9) and adenoid vegetation (J35.2), which were negatively correlated with visibility. Epistaxis (R04.0) was positively correlated with visibility. Some diseases were correlated with multiple weather features, such as enteritis (K52.9), which was correlated with temperature, visibility, humidity and even wind speed at different temporal lags. Several long time-lag correlations also emerged but have no discernible clinical meaning.

### Regression model

Based on the finding that most pediatric diseases were correlated with seasons and various weather factors, a regression analysis that included the temperature, dew point, sea-level pressure, humidity, visibility, wind speed and amount of rainfall was conducted for 200 diseases (we extend the analysis to more diseases in this step). All of these regression models using the monthly average climate feature as predictors can output the monthly disease incidence z-score. The top 10 best regression models, which were evaluated based on the residual deviance, are shown in Supplemental Figure S6. The prediction value fit the true incidence very well in these diseases.

### The pediatric disease co-occurrence network

**Figure 4:**
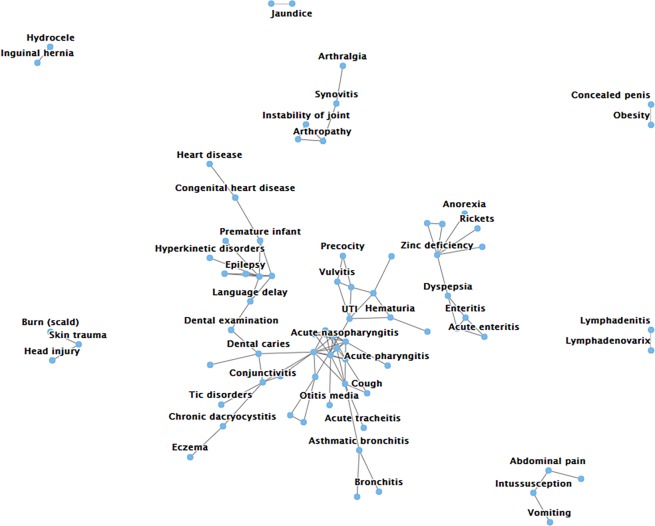
The pediatric disease co-occurrence network. The connected nodes show a cooccurring disease relationships with a log(OR) greater than 2.

A pediatric disease co-occurrence network was generated as shown in Fig. 4. There are some well-known co-occurrence patterns, such as obesity with concealed penises, hydroceles and inguinal hernias, Zinc deficiency and anorexia. However, there are also many interesting novel connections between diseases, such as between tic disorders and conjunctivitis and between dental caries and ametropia. The most closely related diseases involved allergic conjunctivitis, which is connected to many other allergic diseases, such as asthma, allergic rhinitis and inflammation of different parts of the body. It has been suggested that the progression of clinical signs of allergic eczema, asthma, and allergic rhinitis follow each other in time and may cooccur^26^. This comorbidity has been attributed to a common mechanism of altered immunologic mechanisms favoring a systemic response of type 2 helper T-cell cytokines to environmental allergens. Such a disease co-occurrence network will inspire researchers to explore disease relationships and their common pathogenesis. For this reason, we also published a low confidence (log(OR) >1.5) pediatric disease co-occurrence network on PedMap, which will be described below.

### PedMap

Using the results described above, an online pediatric disease map called PedMap was developed and published at http://pedmap.nbscn.org. On PedMap, users select different map styles to explore the age-related, seasonal and climate-related variations in and co-occurrence network for common pediatric diseases. As PedMap was based on a dynamic chart that supports the zooming in and zooming out, users can click on a disease in the map to open a new webpage that provides detailed information about the disease, which includes basic incidence information and incidence z-score curves of the four years accompanied by corresponding weather data, age distributions and comorbidities. The details of PedMap are described in the Supplemental materials “Introduction to PedMap”.

The coefficients of the regression model of the top 100 pediatric diseases were used to develop an online incidence prediction tool that accepts customized climate information to predict the incidence z-score of common pediatric diseases. As different prediction models are characterized by different goodness of fit values, we use the k-means clustering to classify three different prediction confidence levels^27^. The confidence level was labeled with the disease name in the online tools.

## Discussion

PedMap provides an overview of common pediatric diseases, as shown in Supplemental Figure S7. Which clearly shows which diseases will affect children at different stages and in different seasons. The diseases that affect younger children mainly occur in the spring and winter, and diseases that affect older children mostly occur in the summer. The autumn is the safest season for children of all ages. Furthermore, a pediatric disease co-occurrence network was first generated to cover the most common pediatric diseases. Therefore, this knowledge resource and prediction tool could improve the accuracy of the workforce and resource planning of the pediatric department. In China, the government has required medical institutions at all levels to open isolated enteric disease clinics from May to October each year to control enteric infections, such as cholera. However, in this study, the data show that the incidence peak of diarrhea always occurs after October (Supplemental Figure S8). Diarrhea is also classified as an autumn disease instead of a summer disease, as is traditionally assumed^28^. Therefore, it would be wise to keep the enteric disease clinic at the local children’s hospital open until December.

The pediatric disease co-occurrence network contains many connections that have yet been well explained. This network will inspire researchers to further explore the relationships between pediatric diseases and their common pathogenesis. The temporal pattern of co-occurrence will provide more information about the occurrence, development and outcome of diseases.

As PedMap was built based on a local dataset, its applicability to other regions and countries is unknown. Furthermore, in addition to real diseases and climate associations, many social behaviors are factors influencing outpatient visits. For example, summer vacation was the best times to treat phimosis and other chronic conditions, such as obesity, enuresis and short stature in schoolchildren. However, our prediction model did not consider these social factors. Finally, the disease data were obtained from the outpatient department and do not include lower-severity conditions that may not lead to individuals to seek care.

## Conclusion

PedMap provides a convenient and comprehensive view of age-related, seasonal and climate-related variations in and co-occurrence of common pediatric diseases. The prediction tool can help with decision making related to workforce and resource planning in pediatric departments based on weather forecasts. The visualization of epidemiological knowledge of pediatric diseases also helps in the development of evidence-based children’s healthcare programs.

## Supplementary information

Supplemental Documents

Dataset 1

Dataset 2

## Data Availability

All data generated or analyzed during this study are included in this published article and supplementary information files and on the PedMap website (http://pedmpa.nbscn.org).
