# Supplementary material for: PedMap: a pediatric diseases map generated from clinical big data from Hangzhou, China
Source: Sci Rep. 2019 Nov 28;9:17867. doi: 10.1038/s41598-019-54439-w (PMC6883068; doi:10.1038/s41598-019-54439-w)

PedMap: a pediatric diseases map generated from clinical big data from Hangzhou, China

Haomin Li[a#*], PhD; Gang Yu[a#], MSc; Cong Dong[b], BS; Zheng Jia[b], BS; Jiye An[b],PhD; Huilong Duan[b], PhD; Qiang Shu[a*], MD

Summary:
8 supplemental figures, 2 supplemental table, 1 supplemental method, 1 supplemental material "Introduction of PedMap".

1. Supplemental Figures:

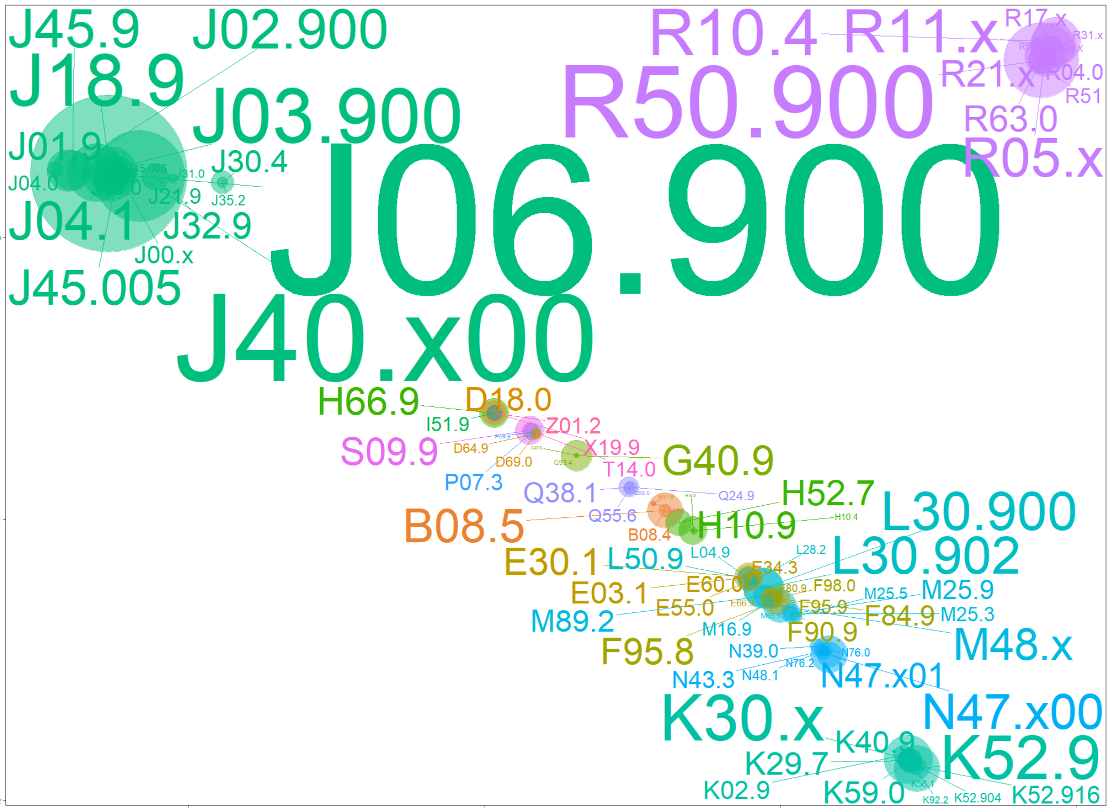

Figure S1. The top 100 most common pediatric diseases. The size of the bubble is based on the incidence per month. The color of the bubble is based on the ICD-10 disease category. The distance between bubbles is based on the semantic distance between different ICD-10 codes but it is no really distance meaning and there is no axis for this graph.

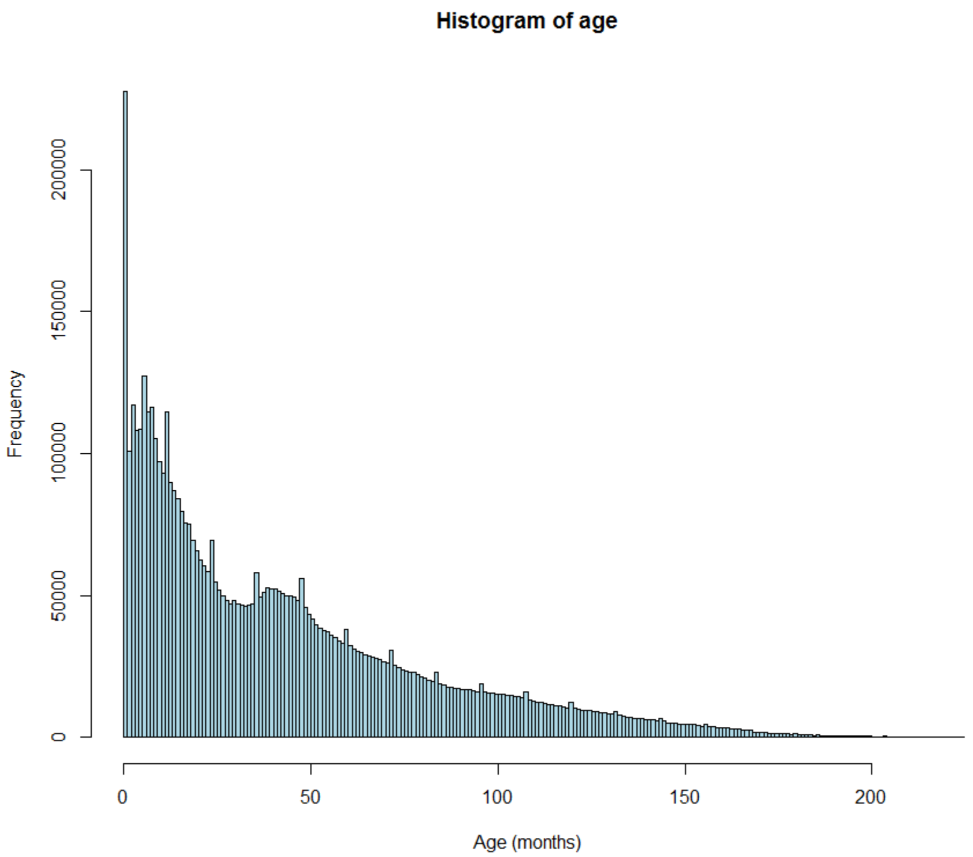

Figure S2. The age distribution of all 5,447,202 outpatient visits.

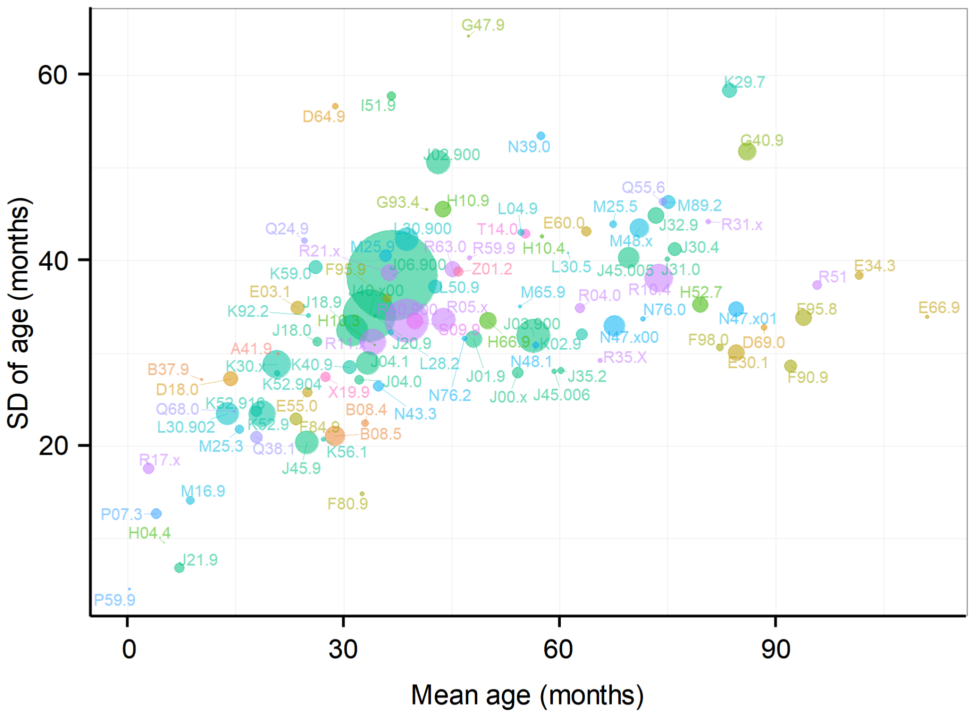

Figure S3. The age features of 100 common pediatric diseases. The size of the bubble is based on the average incidence of disease per month.

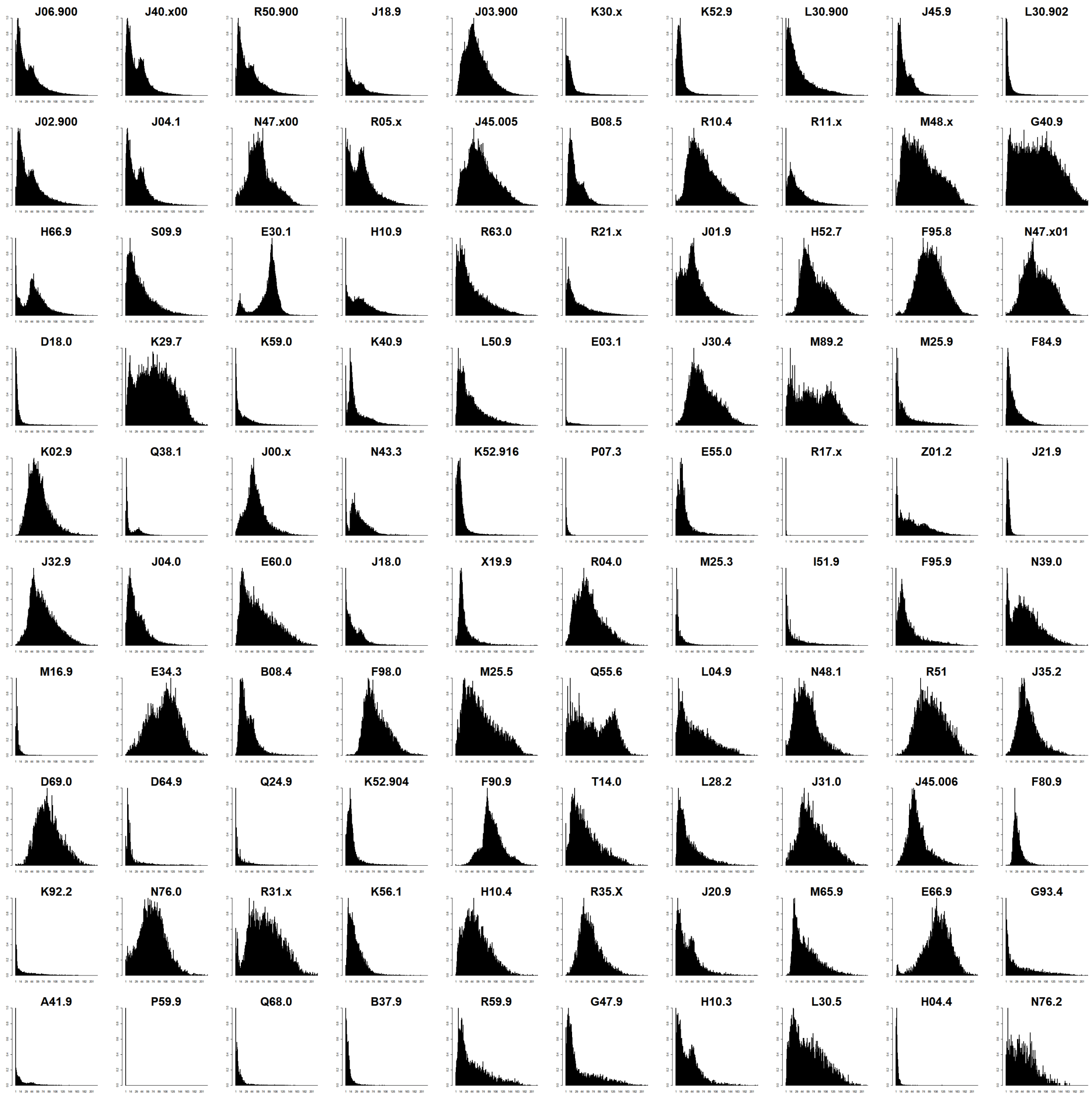

Figure S4. The normalized age distribution of top 100 pediatric diseases. All these histograms were normalized with same X and Y axis scale.

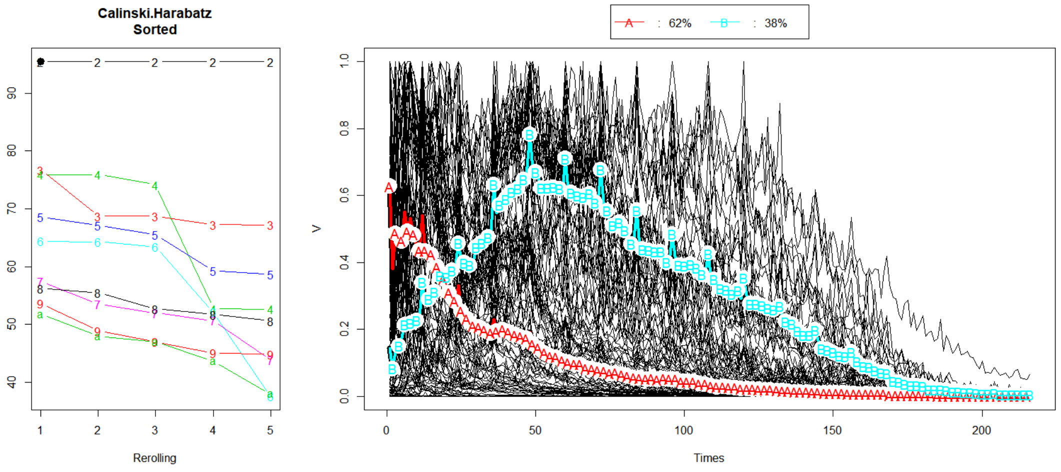

Figure S5. The choice of best k for age pattern clusters. The left part shows the clustering quality criteria (Calinski&Harabatz) of k from 2 to 10. The right part shows "best" k (here the k=2) results on raw trajectories and the mean trajectories of cluster. The kml package provide such a visualization tool to help we choice the "best" k for different studies.

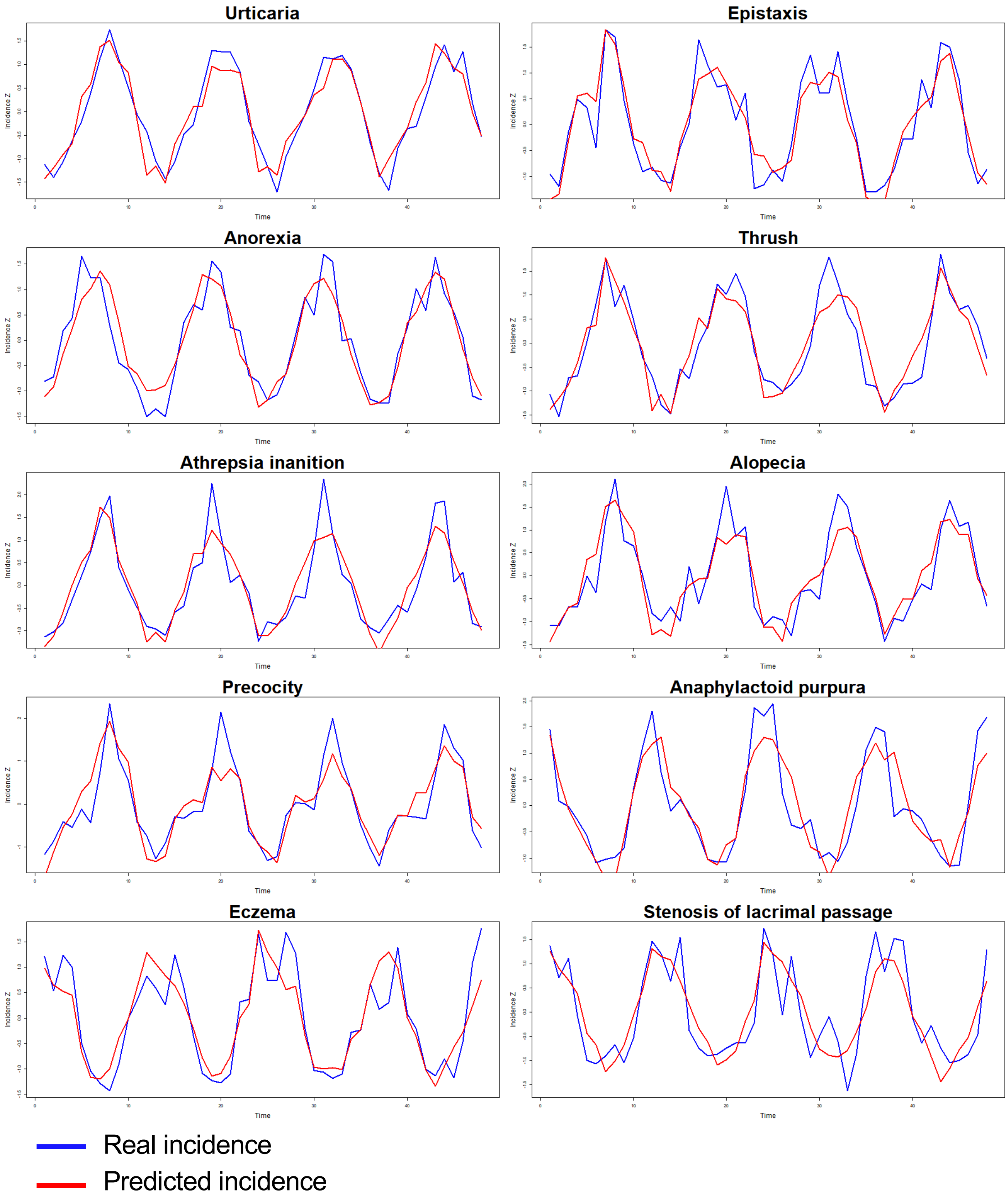

Figure S6. The top 10 best regression model. The real incidence plotted in blue and the predicted incidence based on weather data plotted in red.

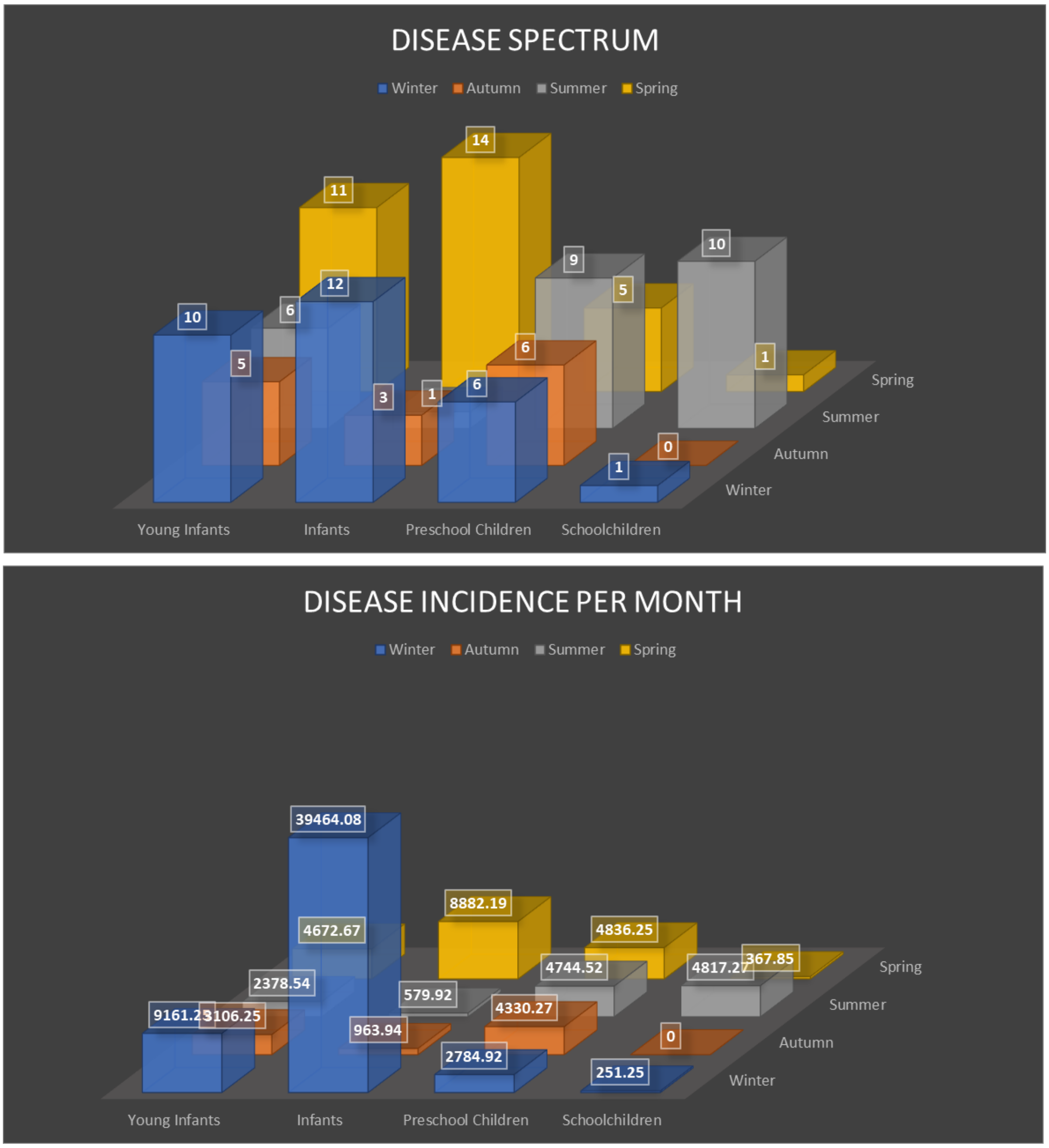

Figure S7. The bird's-eye view of common pediatric diseases distribution in age and season patterns. The top figure shows the number of diseases and the bottom one shows the disease incidences distribution in two patterns.

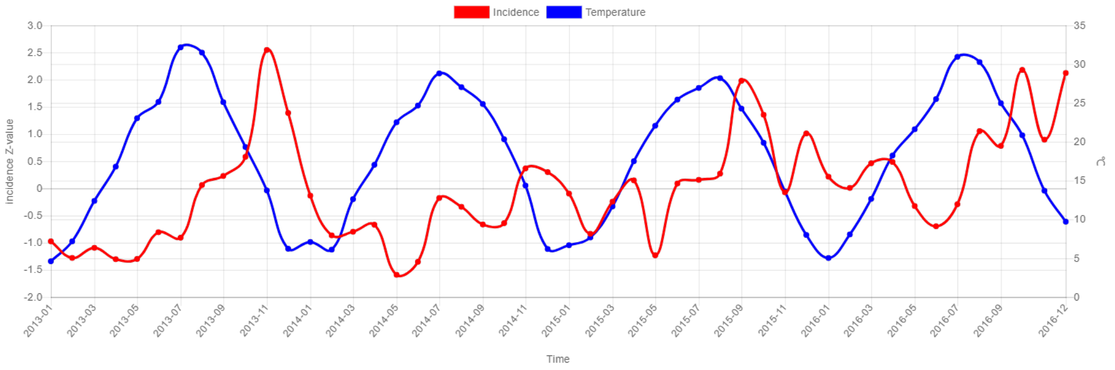

Figure S8. The incidence of diarrhea during 2013-2016. The peaks of diarrhea always show up after October in our local data that will help the administration to adjust current policy and extend the enteric disease clinic until December. Please access it at http://pedmap.nbscn.org/disease/Diarrhea

2. Supplemental Tables:

Table S1. The seasonal and age patterns of common pediatric diseases

Diseases	ICD10	Age (months) Mean±SD	Patient visits/ month	Seasonal Pattern*	Age Pattern#
**AURI**	J06.900	36.74±38.40	20240.19	B	B
**Bronchitis**	J40.x00	33.56±34.00	6933.92	B	B
**FUO**	R50.900	38.77±33.52	4783.52	B	B
**Tonsillitis**	J03.900	56.4±31.84	2875.33	A	C
**Pneumonia**	J18.9	31.23±32.41	2600.56	B	A
**Abdominal pain**	R10.4	73.82±38.10	2068.98	D	C
**Dyspepsia**	K30.x	20.70±28.78	2067.29	D	A
**Enteritis**	K52.9	18.67±23.48	1883.83	B	B
**Vomiting**	R11.x	34.06±31.20	1828.98	B	A
**Acute pharyngitis**	J02.900	43.19±50.58	1543.83	A	B
**Cough**	R05.x	43.89±33.55	1536.69	B	B
**Dermatitis**	L30.900	38.71±42.31	1504.48	A	B
**Asthmatic bronchitis**	J45.9	24.89±20.40	1490.88	B	B
**Acute tracheitis**	J04.1	33.34±28.94	1473.38	B	B
**Eczema**	L30.902	13.90±23.45	1414.90	B	A
**Phimosis**	N47.x00	67.58±32.93	1265.73	C	C
**Asthma**	J45.005	69.61±40.30	1255.46	D	C
**Herpangina**	B08.5	28.77±21.07	1169.52	A	B
**Fracture**	M48.x	71.04±43.51	1046.67	A	C
**Epilepsy**	G40.9	86.01±51.82	940.94	C	D
**Otitis media**	H66.9	50.01±33.50	854.25	B	A
**Acute rhinitis**	J01.9	48.07±31.49	854.25	B	C
**Head injury**	S09.9	39.90±33.48	821.71	A	B
**Precocity**	E30.1	84.59±30.01	813.56	C	A
**Conjunctivitis**	H10.9	43.77±45.57	802.33	B	A
**Tic disorders**	F95.8	93.85±33.88	788.75	C	D
**Rash**	R21.x	36.30±38.71	787.08	A	A
**Nasosinusitis**	J32.9	73.36±44.89	763.67	B	C
**Anorexia**	R63.0	45.19±39.03	744.52	A	B
**Ametropia**	H52.7	79.51±35.25	729.77	C	C
**Redundant prepuce**	N47.x01	84.46±34.78	692.60	C	D
**Hemangioma**	D18.0	14.31±27.22	661.79	A	A
**Gastritis**	K29.7	83.59±58.36	652.40	C	D
**Allergic rhinitis**	J30.4	75.97±41.24	607.75	C	C
**Constipation**	K59.0	26.09±39.28	607.21	A	A
**Inguinal hernia**	K40.9	30.8±28.47	600.63	A	B
**Congenital hypothyroidism**	E03.1	23.61±34.87	587.10	B	A
**Growth retardation**	M89.2	75.10±46.30	583.73	C	C

**Urticaria**	L50.9	42.70±37.17	579.92	C	B
**Eccyliosis**	F84.9	23.42±22.88	521.38	A	B
**Hyperkinetic disorders**	F90.9	92.08±28.62	514.67	C	D
**Ankyloglossia**	Q38.1	17.86±20.95	503.44	A	A
**Arthropathy**	M25.9	35.77±40.57	488.42	C	A
**Saprodontia**	K02.9	63.07±32.04	458.08	A	C
**Acute nasopharyngitis**	J00.x	54.14±27.91	448.02	B	C
**Diarrhea**	K52.916	17.83±23.75	436.17	D	B
**Jaundice**	R17.x	2.87±17.60	427.13	A	A
**Hydrocele**	N43.3	34.79±26.43	415.06	A	A
**Premature infant**	P07.3	4.00±12.68	400.81	A	A
**Rickets**	E55.0	24.94±25.80	394.77	A	B
**Zinc deficiency**	E60.0	63.70±43.16	385.08	C	C
**Epistaxis**	R04.0	62.82±34.87	382.21	C	C
**Dental examination**	Z01.2	45.86±38.81	380.21	C	A
**Pediatric bronchiolitis**	J21.9	7.19±6.83	376.85	B	A
**Burn (scald)**	X19.9	27.42±27.44	375.21	A	B
**Headache**	R51	95.77±37.35	367.85	A	D
**Skin trauma**	T14.0	55.30±42.88	360.46	D	C
**Acute laryngitis**	J04.0	32.20±27.11	356.73	D	B
**Bronchopneumonia**	J18.0	26.33±31.24	353.31	B	A
**Instability of joint**	M25.3	15.53±21.81	340.21	D	A
**Heart disease**	I51.9	36.58±57.78	339.71	C	A
**Convulsion**	F95.9	36.02±35.96	331.02	B	B
**UTI**	N39.0	57.38±53.44	318.75	C	C
**Coxarthropathy**	M16.9	8.69±14.08	315.58	D	A
**Short in stature**	E34.3	101.5±38.38	307.50	C	D
**Concealed penis**	Q55.6	74.30±46.36	304.33	C	C
**HFMD**	B08.4	32.98±22.45	286.10	A	B
**Enuresis**	F98.0	82.29±30.60	283.31	C	D
**Arthralgia**	M25.5	67.46±43.93	282.10	B	C
**Lymphadenitis**	L04.9	54.61±43.08	269.67	A	B
**Balanoposthitis**	N48.1	56.65±30.91	257.13	A	C
**Adenoid vegetation**	J35.2	60.11±28.16	253.40	B	C
**Anaphylactoid purpura**	D69.0	88.41±32.79	251.25	B	D
**Anemia**	D64.9	28.86±56.63	243.44	A	A
**Congenital heart disease**	Q24.9	24.58±42.15	241.25	A	A
**Acute enteritis**	K52.904	20.71±27.88	236.81	B	B
**Papular urticaria**	L28.2	36.52±32.24	227.21	A	B
**Chronic rhinitis**	J31.0	74.95±40.12	223.73	D	C
**Hematuria**	R31.x	80.62±44.17	220.06	C	D
**Vulvitis**	N76.2	46.77±31.59	219.65	A	B
**CVA(Cough variant asthma)**	J45.006	59.24±28.06	217.69	D	C

**Vulvovaginitis**	N76.0	71.55±33.72	216.48	C	D
**Language delay**	F80.9	32.58±14.86	210.29	A	A
**Hematochezia**	K92.2	25.05±34.06	207.67	D	A
**Frequent urination**	R35.X	65.62±29.21	203.96	D	C
**Lymphadenovarix**	R59.9	47.45±40.31	203.52	A	B
**Intussusception**	K56.1	27.15±20.74	200.90	B	B
**Adiposity**	E66.9	111.0±33.94	200.56	C	D
**Allergic Conjunctivitis**	H10.4	57.57±42.62	199.04	A	C
**Acute bronchitis**	J20.9	34.29±34.01	184.75	B	B
**Synovitis**	M65.9	54.47±35.07	183.48	B	C
**Encephalopathy**	G93.4	41.53±45.53	181.96	C	A
**Septicemia**	A41.9	20.88±29.90	181.65	B	A
**Neonatal jaundice**	P59.9	0.21±4.56	175.50	D	A
**Torticollis**	Q68.0	14.76±23.74	175.17	A	A
**Thrush**	B37.9	10.29±27.17	174.69	C	A
**Sleep disorder**	G47.9	47.30±64.24	171.04	D	B
**Acute conjunctivitis**	H10.3	34.33±30.89	168.21	B	B
**Pityriasisalba**	L30.5	61.18±40.81	167.17	C	C
**Chronic dacryocystitis**	H04.4	5.11 ±9.55	161.31	B	A

* Seasonal patterns: A (spring), B (winter), C (summer), D (autumn).

# age patterns: A (young infant), B (infant), C (preschool children) and D (schoolchildren)

Table S2. The correlations among weather features and pediatric diseases. (Cor: pearson correlation coefficient; p: p-value, significant level p<0.05)

**Diseases**	**Temperature**		**Dew point**		**Humidity**		**Sea-level pressure**		**visibility**		**Wind speed**		**rainfall**	
	**Cor**	**p**	**Cor**	**p**	**Cor**	**p**	**Cor**	**p**	**Cor**	**p**	**Cor**	**p**	**Cor**	**p**
**Anaphylactoid purpura**	-0.81	4E-12	-0.78	4.9E-11	-0.11	0.444619	0.86	3.48E-15	-0.56	3.18E-05	-0.49	0.000435	-0.58	1.64E-05
**Anorexia**	0.87	1.16E-15	0.84	6.58E-14	0.12	0.400296	-0.91	1.1E-19	0.54	8.57E-05	0.30	0.036669	0.57	2.77E-05
**Lymphadenitis**	0.78	4.68E-11	0.78	6.61E-11	0.19	0.194783	-0.81	4.1E-12	0.39	0.006469	0.16	0.26713	0.56	3.02E-05
**Pediatric bronchiolitis**	-0.84	5.45E-14	-0.83	4.45E-13	-0.16	0.264003	0.84	1.16E-13	-0.45	0.001164	-0.34	0.017522	-0.54	6.24E-05
**Vomiting**	-0.50	0.000253	-0.48	0.000536	-0.06	0.710024	0.63	1.45E-06	-0.39	0.005919	-0.38	0.007587	-0.54	6.3E-05
**Allergic Conjunctivitis**	0.76	5.13E-10	0.74	1.46E-09	0.16	0.283547	-0.78	4.19E-11	0.41	0.004015	0.16	0.266422	0.54	6.67E-05
**Herpangina**	0.59	1.24E-05	0.59	9.07E-06	0.21	0.147573	-0.65	6.91E-07	0.30	0.036073	-0.06	0.700978	0.54	7.63E-05
**Eczema**	-0.84	8.21E-14	-0.87	1.66E-15	-0.31	0.031513	0.81	2.32E-12	-0.45	0.001238	-0.22	0.137054	-0.54	8.06E-05
**Dermatitis**	0.71	1.15E-08	0.67	1.85E-07	0.02	0.868675	-0.76	3.55E-10	0.42	0.002856	0.24	0.099415	0.54	8.45E-05
**Developmental disorders**	0.78	4.06E-11	0.76	3.25E-10	0.12	0.401499	-0.80	7.16E-12	0.40	0.004993	0.27	0.066243	0.53	0.000124
**Phimosis**	0.76	5.06E-10	0.75	1.12E-09	0.14	0.347465	-0.73	2.73E-09	0.54	8.8E-05	0.28	0.056472	0.52	0.000131
**Bronchopneumonia**	-0.75	7.05E-10	-0.73	4.17E-09	-0.12	0.416856	0.79	3.84E-11	-0.44	0.001835	-0.38	0.007188	-0.52	0.000152
**Pneumonia**	-0.74	1.27E-09	-0.74	1.41E-09	-0.21	0.152365	0.74	2.13E-09	-0.37	0.009369	-0.33	0.020863	-0.51	0.000225
**Asthmatic bronchitis**	-0.67	1.63E-07	-0.62	2.68E-06	0.02	0.880713	0.77	1.33E-10	-0.51	0.000196	-0.44	0.001905	-0.50	0.000279
**Bronchitis**	-0.64	7.81E-07	-0.62	2.7E-06	-0.08	0.595984	0.72	1.13E-08	-0.41	0.003555	-0.37	0.009337	-0.49	0.000452
**Acute tracheitis**	-0.74	2.48E-09	-0.69	6.03E-08	-0.01	0.93905	0.78	5.64E-11	-0.55	5.37E-05	-0.38	0.007413	-0.48	0.000527

**Otitis media**	-0.59	8.8E-06	-0.58	1.49E-05	-0.12	0.433639	0.69	6.92E-08	-0.43	0.002408	-0.32	0.028023	-0.48	0.000576
**Acute bronchitis**	-0.52	0.000132	-0.52	0.000141	-0.14	0.341504	0.54	6.59E-05	-0.32	0.024382	-0.34	0.016512	-0.45	0.001193
**Zinc deficiency**	0.80	8.63E-12	0.77	2.09E-10	0.07	0.62838	-0.74	2.1E-09	0.44	0.001934	0.29	0.046386	0.45	0.00147
**Headache**	0.55	4.6E-05	0.53	9.2E-05	0.08	0.584208	-0.60	8.12E-06	0.34	0.017564	-0.04	0.810524	0.44	0.001928
**Epistaxis**	0.86	3.12E-15	0.78	9.9E-11	-0.12	0.401811	-0.87	4.38E-16	0.64	1.06E-06	0.45	0.001298	0.43	0.002086
**Septicemia**	-0.46	0.001078	-0.48	0.000625	-0.18	0.223656	0.52	0.000138	-0.27	0.064768	-0.26	0.069328	-0.43	0.002228
**Rickets**	0.59	9.16E-06	0.52	0.000135	-0.08	0.585781	-0.68	8.7E-08	0.40	0.005104	0.28	0.051912	0.43	0.002381
**Anemia**	0.75	6.82E-10	0.72	6.83E-09	0.09	0.53984	-0.72	7.09E-09	0.41	0.00343	0.29	0.04941	0.42	0.002659
**Acute rhinitis**	-0.60	5.61E-06	-0.57	2.13E-05	-0.03	0.832773	0.64	8.61E-07	-0.50	0.00029	-0.42	0.002612	-0.42	0.00278
**Ametropia**	0.68	8.85E-08	0.66	3.32E-07	0.06	0.679097	-0.67	2.08E-07	0.53	9.74E-05	0.29	0.047122	0.42	0.003066
**HFMD**	0.47	0.000845	0.50	0.000318	0.28	0.055883	-0.50	0.00026	0.16	0.268712	-0.15	0.324955	0.41	0.003593
**Precocity**	0.83	2.06E-13	0.81	4.67E-12	0.09	0.557598	-0.72	6.61E-09	0.52	0.00013	0.54	6.52E-05	0.40	0.005029
**Rash**	0.64	8.7E-07	0.62	2.42E-06	0.10	0.497118	-0.64	1.03E-06	0.30	0.037182	-0.03	0.822719	0.40	0.005083
**Lymphadenovarix**	0.73	5.17E-09	0.69	6.18E-08	0.06	0.677893	-0.75	1.13E-09	0.48	0.000599	0.17	0.250665	0.40	0.005126
**Enteritis**	-0.42	0.002921	-0.45	0.001259	-0.24	0.094281	0.52	0.000178	-0.25	0.088568	-0.14	0.347924	-0.40	0.005351
**Arthralgia**	-0.40	0.004974	-0.39	0.006657	-0.12	0.421887	0.50	0.000307	-0.23	0.116669	0.03	0.832368	-0.40	0.005387
**Growth retardation**	0.75	8.75E-10	0.69	4.25E-08	-0.05	0.76003	-0.74	1.44E-09	0.61	4.6E-06	0.47	0.000713	0.39	0.005646
**Urticaria**	0.88	2.95E-16	0.88	1.92E-16	0.21	0.145599	-0.74	1.89E-09	0.47	0.000809	0.34	0.019271	0.39	0.00566
**Acute pharyngitis**	0.48	0.000614	0.50	0.000288	0.24	0.098439	-0.51	0.000202	0.17	0.260057	-0.15	0.29508	0.39	0.005864
**Congenital heart disease**	0.55	4.87E-05	0.58	1.46E-05	0.24	0.096752	-0.47	0.000667	0.21	0.161733	0.01	0.938334	0.39	0.006644
**Papular urticaria**	0.66	2.7E-07	0.72	9.48E-09	0.39	0.005563	-0.60	6.73E-06	0.20	0.173779	0.06	0.66284	0.38	0.007147
**Thrush**	0.88	8.51E-17	0.87	2.15E-15	0.14	0.33683	-0.78	5.42E-11	0.54	8.42E-05	0.32	0.029142	0.38	0.007257
**Cough**	-0.34	0.017317	-0.35	0.013844	-0.12	0.411069	0.38	0.007189	-0.29	0.042354	-0.41	0.004181	-0.38	0.007479
**Hydrocele**	0.82	1.73E-12	0.79	1.68E-11	0.11	0.452739	-0.71	1.14E-08	0.47	0.000824	0.26	0.078673	0.38	0.008156
**Chronic dacryocystitis**	-0.48	0.000631	-0.52	0.000144	-0.30	0.036625	0.43	0.002076	-0.17	0.254756	-0.06	0.671837	-0.37	0.009691
**Acute laryngitis**	-0.15	0.317752	-0.14	0.332458	-0.07	0.620777	0.32	0.024507	-0.09	0.542502	0.00	0.992676	-0.36	0.012013
**Nasosinusitis**	-0.55	4.35E-05	-0.56	4.22E-05	-0.14	0.348633	0.55	4.64E-05	-0.39	0.006801	-0.48	0.000551	-0.36	0.012517
**Concealed penis**	0.71	2.14E-08	0.66	4.22E-07	-0.04	0.802778	-0.70	4.12E-08	0.59	1.19E-05	0.34	0.017017	0.35	0.014266
**Acute enteritis**	-0.40	0.004952	-0.40	0.004693	-0.13	0.379859	0.49	0.000389	-0.28	0.051253	-0.20	0.162366	-0.35	0.014735
**Head injury**	0.64	7.71E-07	0.63	1.68E-06	0.12	0.42898	-0.59	1.01E-05	0.35	0.014871	0.33	0.022222	0.35	0.015694
**Pityriasisalba**	0.73	4.21E-09	0.73	4.67E-09	0.16	0.27127	-0.61	4.55E-06	0.41	0.003921	0.19	0.195475	0.34	0.017328
**Adiposity**	0.73	4.61E-09	0.69	7.26E-08	0.00	0.976271	-0.67	2.5E-07	0.58	1.82E-05	0.43	0.002582	0.33	0.021817
**Arthropathy**	0.59	9.83E-06	0.58	1.5E-05	0.09	0.562881	-0.52	0.000161	0.37	0.010009	0.35	0.014914	0.33	0.022908
**Tonsillitis**	0.17	0.240082	0.16	0.265919	0.05	0.741135	-0.32	0.026626	0.05	0.719878	-0.21	0.142404	0.33	0.024063
**Heart disease**	0.62	3.04E-06	0.56	3.97E-05	-0.08	0.599844	-0.54	8.17E-05	0.45	0.001484	0.36	0.011033	0.32	0.024927
**Fracture**	0.79	3.85E-11	0.75	5.72E-10	0.08	0.604478	-0.68	1.06E-07	0.38	0.007087	0.27	0.067727	0.32	0.025361
**Sleep disorder**	-0.11	0.466635	-0.13	0.381219	-0.16	0.287885	0.28	0.054363	-0.03	0.849475	0.18	0.210801	-0.32	0.025627
**Dental examination**	0.58	1.9E-05	0.49	0.000417	-0.18	0.231943	-0.63	1.74E-06	0.52	0.000132	0.42	0.002901	0.32	0.028032
**Allergic rhinitis**	0.75	1.19E-09	0.74	1.35E-09	0.16	0.28374	-0.60	5.84E-06	0.43	0.002347	0.25	0.084048	0.32	0.028758
**Congenital hypothyroidism**	-0.46	0.000994	-0.47	0.00077	-0.18	0.208871	0.40	0.004372	-0.31	0.032799	-0.20	0.175992	-0.30	0.036053
**Torticollis**	0.80	8.62E-12	0.78	4.15E-11	0.14	0.346189	-0.70	3.11E-08	0.36	0.010987	0.22	0.126654	0.30	0.038625
**UTI**	0.73	4.87E-09	0.69	7.06E-08	0.00	0.974279	-0.62	2.32E-06	0.49	0.000368	0.35	0.014333	0.28	0.051272
**Diarrhea**	-0.10	0.480477	-0.09	0.544997	0.00	0.986632	0.29	0.049372	-0.16	0.275953	-0.05	0.729842	-0.28	0.057294

**Convulsion**	-0.35	0.013615	-0.39	0.006355	-0.24	0.10607	0.35	0.015371	-0.17	0.242698	-0.22	0.129145	-0.27	0.062776
**Vulvovaginitis**	0.46	0.001006	0.47	0.000827	0.14	0.357537	-0.38	0.008545	0.18	0.227198	0.13	0.365754	0.27	0.066642
**Language delay**	0.68	1.16E-07	0.66	2.72E-07	0.11	0.473965	-0.55	5.01E-05	0.35	0.015426	0.37	0.009282	0.26	0.07868
**Acute nasopharyngitis**	-0.34	0.019355	-0.33	0.022643	-0.04	0.793953	0.42	0.002836	-0.31	0.029216	-0.26	0.07878	-0.25	0.086204
**Acute conjunctivitis**	-0.64	1.2E-06	-0.68	1.18E-07	-0.31	0.033997	0.47	0.000737	-0.28	0.050632	-0.18	0.233319	-0.24	0.095294
**Encephalopathy**	0.63	1.49E-06	0.56	3.75E-05	-0.14	0.336473	-0.53	9.93E-05	0.40	0.004486	0.41	0.004202	0.24	0.096787
**Asthma**	-0.08	0.605826	-0.03	0.833335	0.13	0.360998	0.26	0.080132	-0.21	0.147959	-0.35	0.014945	-0.24	0.097925
**Saprodontia**	0.38	0.006915	0.37	0.009557	0.06	0.690817	-0.38	0.007909	0.17	0.238931	-0.04	0.766347	0.24	0.104493
**Short in stature**	0.59	8.89E-06	0.52	0.000133	-0.14	0.351859	-0.59	9.17E-06	0.60	7.52E-06	0.44	0.001663	0.24	0.105511
**Adenoid vegetation**	-0.57	2.76E-05	-0.61	3.79E-06	-0.31	0.032995	0.39	0.005566	-0.21	0.15146	-0.20	0.17868	-0.24	0.107456
**Constipation**	0.18	0.216136	0.16	0.283645	-0.01	0.965253	-0.24	0.101481	0.03	0.822008	-0.02	0.899748	0.23	0.120656
**Tic disorders**	0.48	0.000604	0.46	0.001038	0.04	0.784156	-0.36	0.010909	0.25	0.081828	0.33	0.020294	0.21	0.148254
**AURI**	-0.11	0.472653	-0.13	0.375474	-0.13	0.368491	0.17	0.239577	-0.04	0.776761	-0.18	0.23315	-0.20	0.162795
**Hematochezia**	-0.02	0.885891	0.04	0.782858	0.23	0.109249	0.15	0.317429	-0.24	0.103304	-0.23	0.111305	-0.20	0.175505
**Neonatal jaundice**	0.36	0.012745	0.38	0.00833	0.20	0.17398	-0.25	0.081552	0.01	0.930289	-0.02	0.906538	0.20	0.175917
**Hemangioma**	0.32	0.029146	0.25	0.086826	-0.16	0.269741	-0.34	0.016409	0.27	0.065997	0.34	0.019209	0.20	0.178893
**Vulvitis**	0.35	0.013513	0.38	0.007571	0.20	0.176549	-0.36	0.011289	0.10	0.489006	-0.14	0.355543	0.19	0.195879
**Synovitis**	-0.19	0.185519	-0.21	0.147043	-0.12	0.422301	0.26	0.071684	-0.22	0.135811	-0.08	0.592119	-0.18	0.229274
**Hematuria**	0.62	2.95E-06	0.55	4.51E-05	-0.12	0.416401	-0.53	0.000105	0.45	0.001254	0.20	0.177279	0.17	0.240615
**Balanoposthitis**	0.23	0.112503	0.24	0.093911	0.09	0.528224	-0.29	0.04321	0.13	0.380929	-0.16	0.278626	0.17	0.252838
**Hyperkinetic disorders**	0.65	5.16E-07	0.64	8.93E-07	0.10	0.479166	-0.50	0.000342	0.47	0.000726	0.41	0.003584	0.16	0.264196
**Intussusception**	-0.09	0.562032	-0.13	0.381744	-0.19	0.207813	0.08	0.566172	-0.07	0.639656	-0.09	0.539708	-0.16	0.279269
**Ankyloglossia**	0.23	0.119449	0.17	0.237118	-0.12	0.422003	-0.27	0.068423	0.12	0.412334	0.13	0.377904	0.14	0.347375
**Redundant prepuce**	0.58	1.77E-05	0.52	0.000159	-0.09	0.523822	-0.54	7.57E-05	0.49	0.000461	0.14	0.345547	0.13	0.369126
**Skin trauma**	0.55	4.78E-05	0.53	0.000111	0.05	0.731194	-0.41	0.004261	0.32	0.024378	0.23	0.116032	0.13	0.369417
**Enuresis**	0.16	0.283631	0.14	0.3534	-0.09	0.523899	-0.04	0.782584	0.21	0.158114	0.11	0.470417	-0.12	0.405573
**Epilepsy**	0.07	0.653747	0.02	0.87795	-0.19	0.205774	-0.03	0.843398	0.20	0.174772	0.11	0.475502	-0.11	0.438203
**Instability of joint**	0.15	0.3076	0.16	0.279176	0.04	0.773977	0.01	0.961308	0.02	0.914934	-0.01	0.937478	-0.11	0.44046
**Jaundice**	0.51	0.000189	0.51	0.000206	0.09	0.558805	-0.39	0.005667	0.26	0.07686	0.06	0.687004	0.11	0.460811
**Chronic rhinitis**	0.10	0.480424	0.09	0.559456	-0.05	0.719178	0.01	0.963834	0.08	0.578921	0.04	0.786115	-0.10	0.480031
**FUO**	-0.03	0.835473	-0.05	0.725525	-0.07	0.618235	-0.04	0.76908	-0.07	0.624268	-0.31	0.032482	0.10	0.510315
**Coxarthropathy**	0.20	0.18084	0.17	0.258617	-0.06	0.673909	-0.08	0.594207	0.10	0.480172	0.14	0.347112	0.09	0.533435
**Dyspepsia**	0.55	6.19E-05	0.57	2.15E-05	0.22	0.135733	-0.33	0.021783	0.19	0.193627	0.09	0.560291	0.08	0.575644
**Abdominal pain**	0.27	0.065183	0.23	0.112217	-0.09	0.553056	-0.15	0.318529	0.15	0.306756	0.10	0.48683	-0.07	0.640111
**Burn (scald)**	0.14	0.331041	0.07	0.636513	-0.24	0.097557	-0.11	0.440592	0.08	0.577609	0.20	0.183579	0.05	0.717978
**CVA**	0.28	0.055327	0.30	0.040788	0.11	0.462803	-0.14	0.327632	0.13	0.387792	-0.01	0.938386	0.04	0.8085
**Frequent urination**	0.28	0.051435	0.27	0.061848	-0.01	0.926968	-0.09	0.5573	0.22	0.127469	0.35	0.013865	-0.03	0.826225
**Inguinal hernia**	0.31	0.029212	0.31	0.03267	0.04	0.803412	-0.33	0.022284	0.18	0.230748	0.11	0.465039	0.03	0.863319
**Gastritis**	0.37	0.009734	0.37	0.009476	0.07	0.640673	-0.21	0.147064	0.19	0.187095	0.15	0.295814	0.02	0.893959
**Conjunctivitis**	0.00	0.992433	-0.02	0.909347	-0.10	0.513113	-0.04	0.801326	0.07	0.624723	0.03	0.831987	0.01	0.929249
**Premature infant**	0.41	0.00382	0.35	0.013547	-0.14	0.354031	-0.29	0.047364	0.27	0.064724	0.24	0.095102	-0.01	0.94211

3.Supplemental Methods

3.1 The data preprocessing and clustering of longitudinal data

Regarding the data, we collected the diagnosis of all patients who first visited a children's hospital for a certain condition between January 1, 2013, and December 31, 2016. There were total of 5 447 202 outpatient visits of 2 189 868 unique patients with 6 433 different diagnoses terms during this period. Then, we selected 100 most common pediatric diseases based on the monthly average incidence of diseases per month. The 48 monthly disease incidences of 100 most common diseases were used to construct the disease incidence data. The corresponding ages in months when patients visited were also collected for each outpatient visit. For each disease, using bin width of 1 month we counted how many patients fall into each bin to construct an age distribution histogram. Both the normalized age histograms over 216 months (18 years) and incidence z-scores over 48 months are type of longitudinal data. Clustering longitudinal data is key method used in this study. We will describe the detail of this method in the following several paragraphs.

All these data were normalized before the clustering. For the disease incidence data, disease incidences of each month for specific disease were normalized as z-scores as shown in following formula, which are the signed fractional numbers of standard deviations by which the diseases incidence is above or below the overall mean across the 48 months.

$$z_i = \frac{x_i - \bar{x}}{sd}$$

Where x_i is the disease incidences in the ith month;
$\bar{x}$ is mean of disease incidences across the 48 months;
sd is the standard deviation of disease incidences across the 48 months.

After such a pre-processing, the seasons fluctuation of each disease will be normalized to a reasonable range from -3 to 3. The age distribution data was normalized in a straightforward

way that maps all values from [0,maximum] into the range [0,1].

The normalized two datasets were released as appendixes “age distribution.csv” and “normalized disease incidence.csv”. In the “age distribution.csv” file, the first column is the disease name and the other 216 columns represent the age of patients in month. 216 months (equal 18 years) covered all the patient that allow accepted by a Children’s hospital in China. The data was normalized so the age distribution was mapping in a range from 0 to 1. This could align different diseases with various incidence. In the “normalized disease incidence.csv” file, the first column is the disease name and the other 48 columns corresponding disease incidence in 48 months (from Jan 2013 to Dec 2016).

We used the kml package in R (version 3.4.0) to cluster longitudinal data. This package is an implementation of k-means clustering for longitudinal data (or trajectories). For this study, the disease incidence z-scores in 48 months of 100 diseases were transformed into an object ClusterLongData at first. Once an object of class ClusterLongData has been created, the algorithm kml can be run to build a Partition. An object of class Partition is a partition of trajectories into subgroups. It also contains some information like the percentage of trajectories contained in each group or some quality criterion. kml is a "hill-climbing" algorithm. The specificity of this kind of algorithm is that it always converges towards a maximum, but one cannot know whether it is a local or a global maximum. It offers no guarantee of optimality. To maximize one's chances of getting a quality Partition, it is better to run the hill climbing algorithm several times, then to choose the best solution. By default, kml executes the hill climbing algorithm 20 times and chooses the Partition maximizing the determinant of the matrix between. Likewise, it is not possible to know beforehand the optimum number of

clusters. In the study, to identify the four seasons associated with pediatric diseases, k=4 was also used to cluster the 100 most common pediatric diseases incidences data, as shown in Figure 2B. To clustering the age patterns, we used the kml package to cluster these 100 age distributions using a k value ranging from 2 to 10 and reran the model 5 times with different initial conditions. The kml package provides a graphical interface for choosing the 'best' number of clusters.

Determining the optimal number of clusters in a data set and making it can be easily interpreted is a fundamental issue in partitioning clustering, such as k-means clustering, which requires the user to specify the number of clusters k to be generated. In this study, we use the k=4 to clustering the seasonal pattern in order to interpret the pattern more easily and can directly mapping it to the four seasons. As shown in the lower half of Figure 2B, the four clusters A, B, C, D which with mean trajectories peaks at different time in each year. To make these clusters meaningful and self-interpretable, we used four seasons (spring, summer, autumn and winter) to name the four clusters based on corresponding seasons of trajectories peaks. Four seasons in the sense of meteorology in Hangzhou is based on the following graph. The four seasons are not the average division of the year. The spring starts from the early March and ends at middle of June. The summer follows it and end at early September. Then the autumn starts and ends at early of December. The winter until the beginning of March of the second year. The four clusters maybe not perfectly match with corresponding seasons in all four years. But it still can be interpreted to four seasons: Cluster A (spring), Cluster B (winter), Cluster C (summer), Cluster D (autumn).

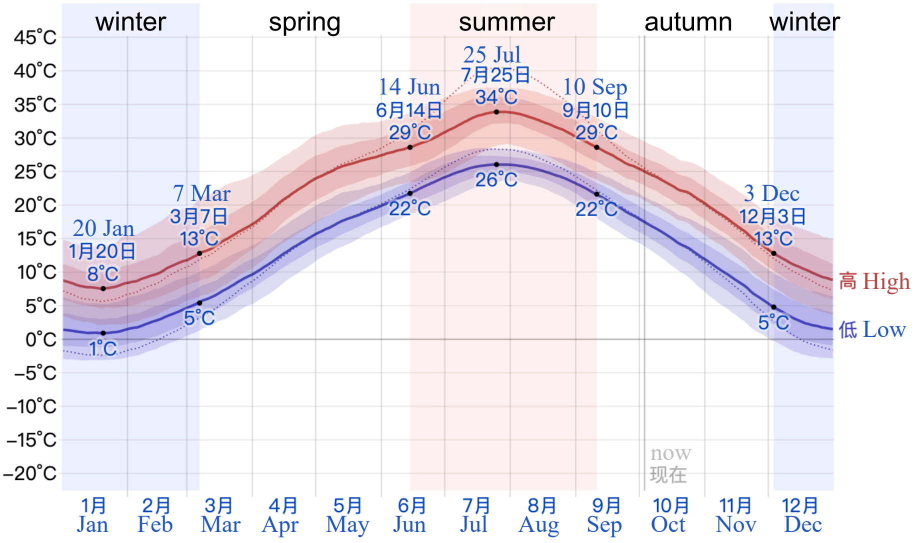

Figure. Four seasons in the sense of meteorology in Hangzhou.

The longitudinal clustering analysis results were shown in Figure 1A and Figure 2B. In the upper part of the figure, each disease was show as a line. The color of line depend on the clustering result will help us to recognize the fluctuation pattern of two datasets.

3.2 The distance among ICD-10 coded pediatric diseases

ICD-10 code as a hierarchy, taxonomy of the diseases contains the information to evaluate the semantic similarity between two diseases. Here the widely used Information content (IC) approach was used to assess the semantic similarity between two ICD-10 codes [1]. For example, a and b are two ICD-10 codes, c is defined as the least common ancestor (or superconcept) of two codes a and b in the ICD-10 hierarchy. The IC of a can be calculated by computing the count of taxonomical leaves of a concept's hyponym tree ($|leaves(a)|$) corresponding to its degree of generality and the number of taxonomical subsumers ($|subsumers(a)|$) representing its degree of concreteness [2].

$$IC(a) = -\log\left(\frac{\frac{|leaves(a)|}{|subsumers(a)|}+1}{|leaves(root)|+1}\right) \qquad (1)$$

The similarity of two ICD codes is defined as:

$$CSim(a,b) = \frac{IC(a)+IC(b)-2IC(c)}{IC(a)+IC(b)} \qquad (2)$$

The top 100 pediatric diseases distance matrix were calculated based on above two formula.

The distance matrix was mapping to 2D space to show the relative distance among different

diseases using multidimensional scaling. The distance on the 2D space reflected the distance in the diseases space. The figure S1 and some online chart on PedMap using these ICD distance to show relationships among diseases.

Reference:

1. Harispe S, Sanchez D, Ranwez D, et al. A framework for unifying ontology-based semantic similarity measures: A study in the biomedical domain. J Biomed Inform 2014; 48:38-53.
2. Sánchez D, Batet M, Isern D. Ontology-based information content computation. Knowl Based Syst 2010; 24:297-303.

4. Introduction to PedMap

1. Access to PedMap

PedMap was published on the internet and can be accessed at http://pedmap.nbscn.org using any web browser. We prefer Google Chrome for the best performance.

The home page of PedMap provided a search box and three major navigation buttons at the top. The “PreMap” button will direct users to the PedMap section on the home page. The “Predict” button will redirect to the Poisson regression model prediction tool. The “About PedMap” button will provide an introduction to PedMap. The search box with autocomplete feature will help user to select pediatric disease and the “Search” button will open the selected disease page.

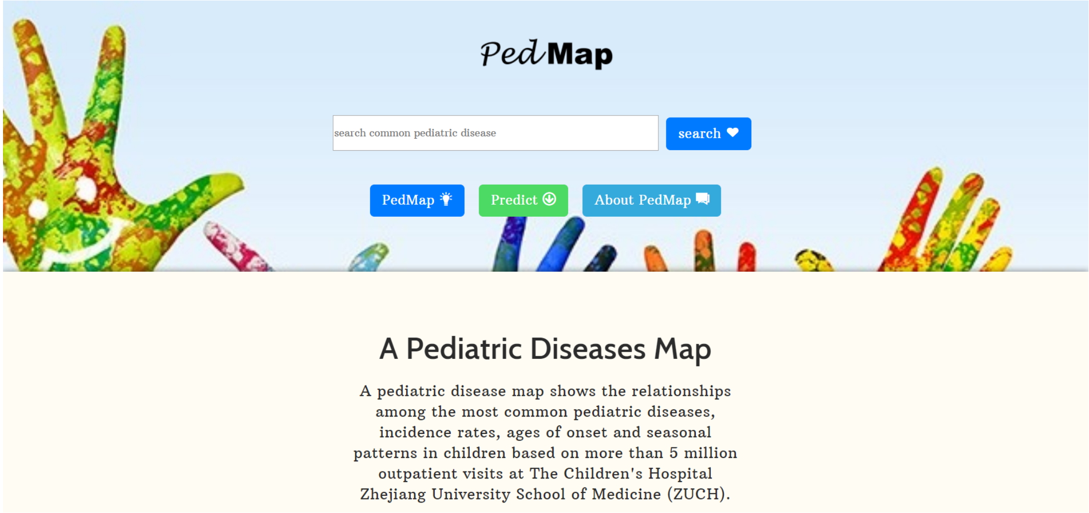

2. PedMap: age-related, seasonal and climate-related variations

PedMap provides 6 different map styles for users to explore the most common pediatric diseases based on age-related, seasonal and climate-related variations. Users can select different styles and refresh PedMap at any time.

1) Based on the Age Relationship

The default map style is based on the age relationship. The x-axis shows the mean age of disease onset, and the y-axis shows the standard deviation of the age.

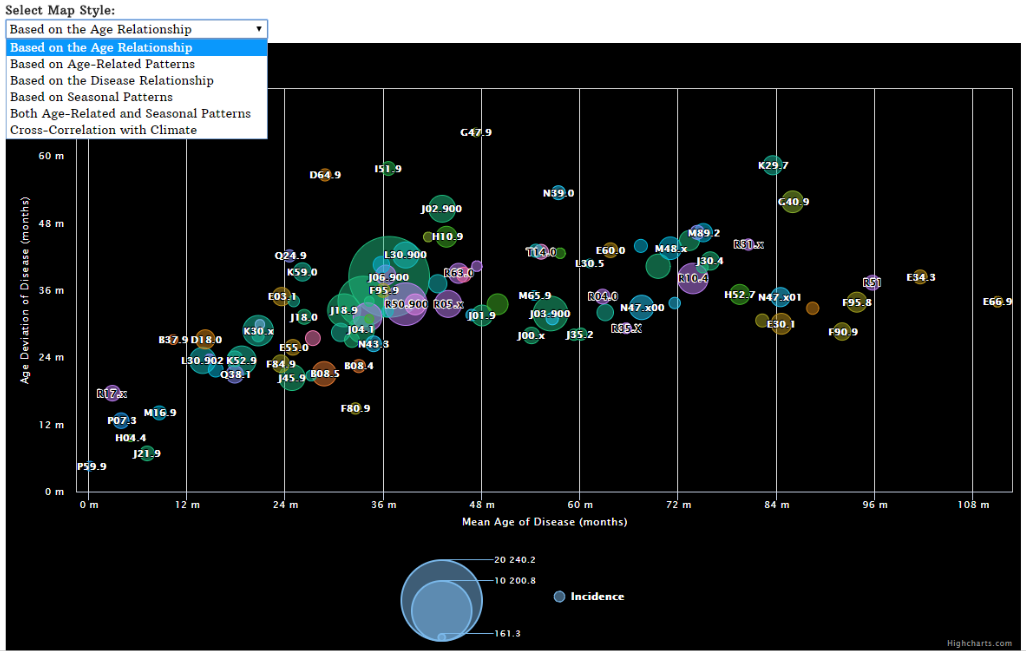

2) Based on Age-related Patterns

The x-axis shows the mean age of diseases. The y-axis shows categories of age patterns: young infants, infants, preschool children and schoolchildren.

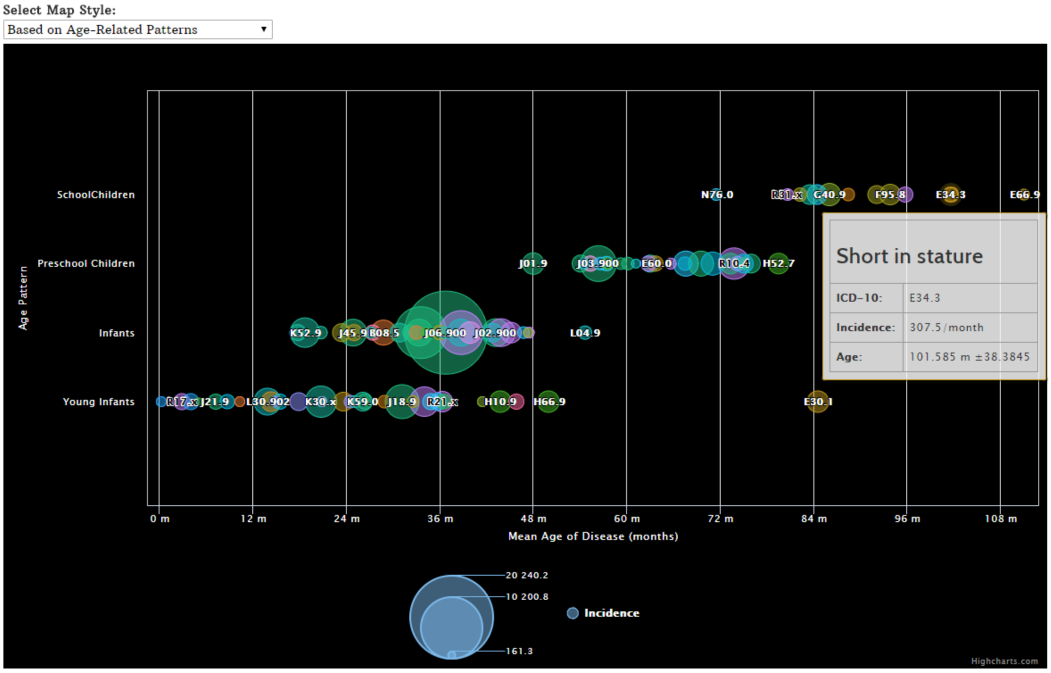

3) Based on Disease Relationship

The common pediatric diseases were plotted in 2D space based on distances among them. The plot is similar to that shown in Figure S1 in this paper.

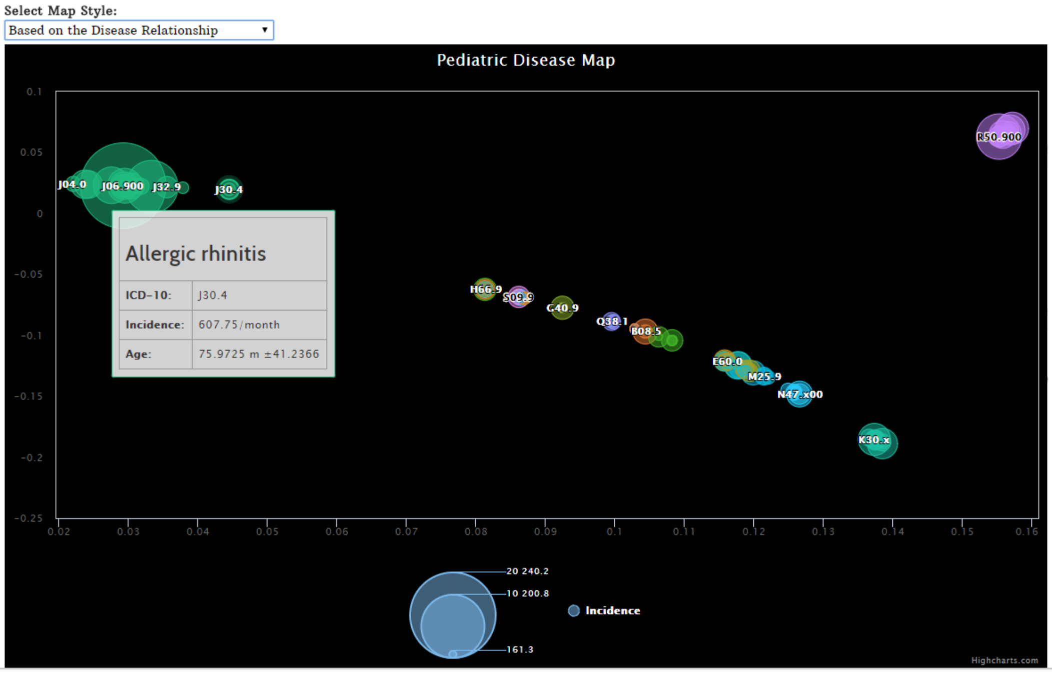

4) Based on Seasonal Patterns

In this style, the y-axis is changed to seasonal pattern categories: spring, summer, autumn and winter.

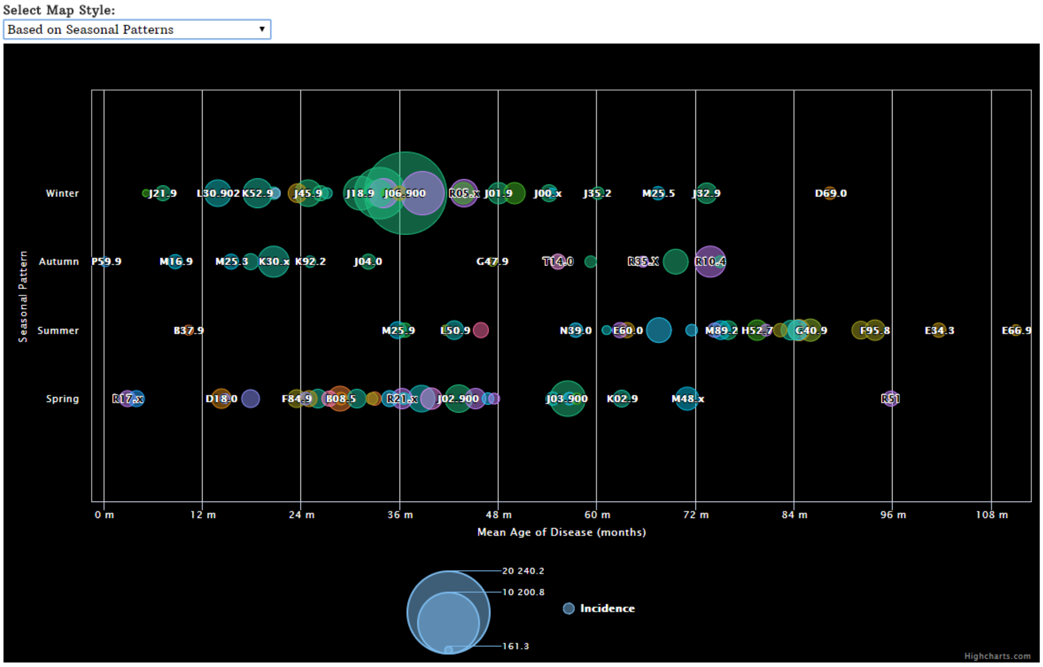

5) Both Age-Related and Seasonal Patterns

The x-axis shows the age patterns and the y-axis shows the seasonal patterns. It provides 4x4 grids to show the distributions of pediatric diseases in two patterns.

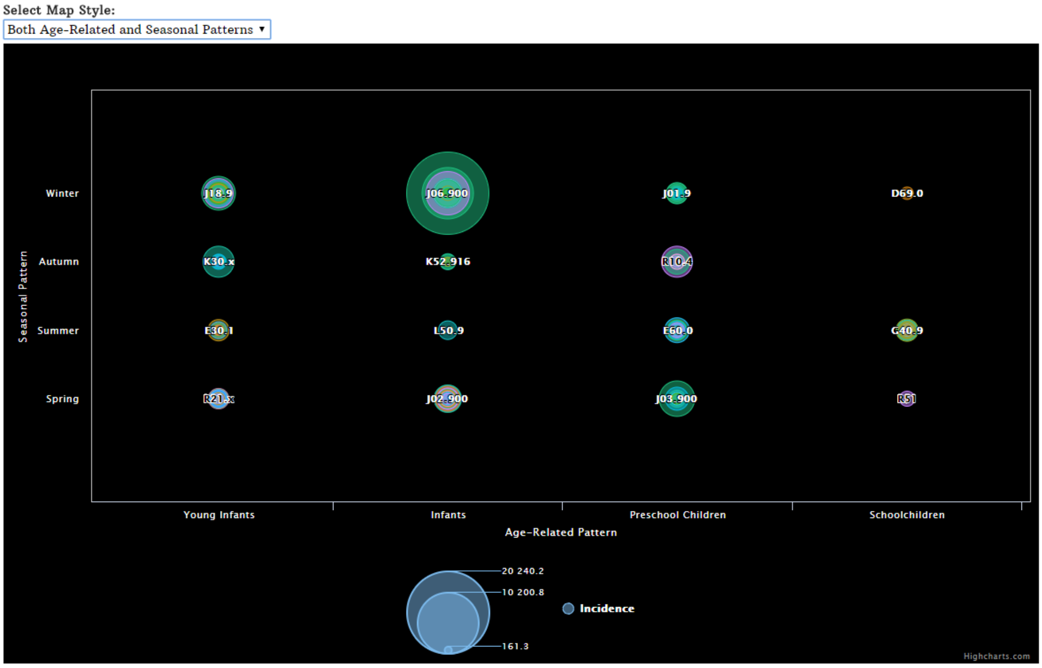

6) Cross-Correlation with Climate

This style has different x- and y-axes. The y-axis shows the correlation R values between disease incidence and weather features (the different color of the bubble indicates a different weather feature). The x-axis shows the temporal lag of the correlation in weeks. This map is similar to that shown in Figure 3B.

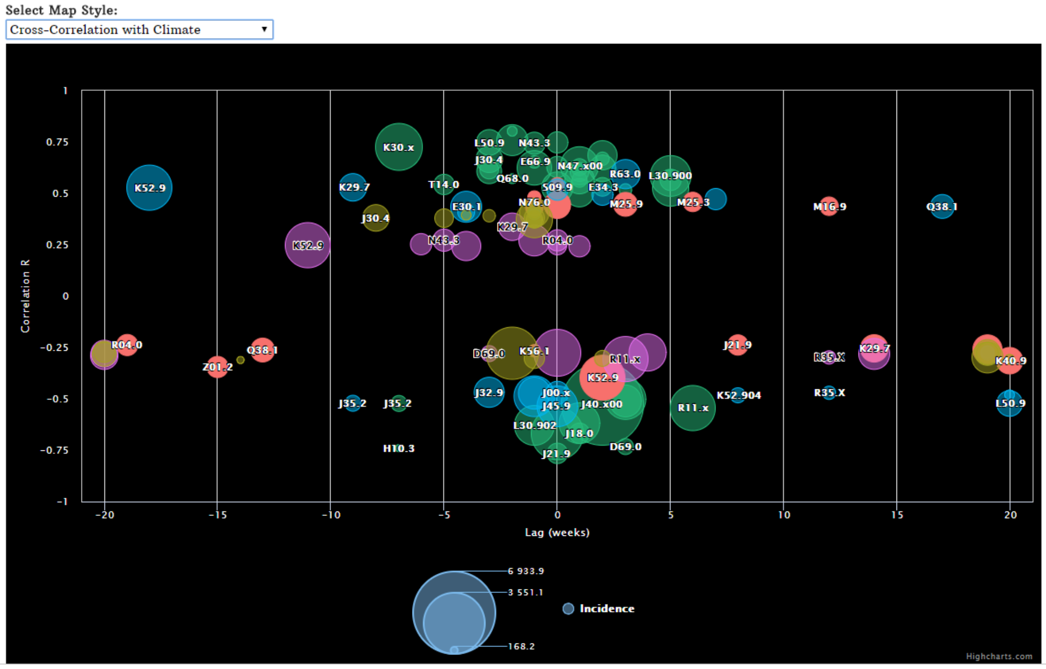

In every map style, users can zoom in through selecting an area using the mouse. A tip will appear when one hovers the mouse over a bubble to show detailed information about the disease. Clicking on the bubble will redirect users to a disease page, which will be described later.

3. PedMap: Pediatric diseases co-occurrence network

The pediatric diseases co-occurrence network will be shown on the home page on default. The name of each node in the network will be show up when the mouse hover on the node. Clicking the node will redirect to the disease page. The node can be dragged and moved. It also proved an option to load a co-occurrence network with low confidence through select it on the left top corner.

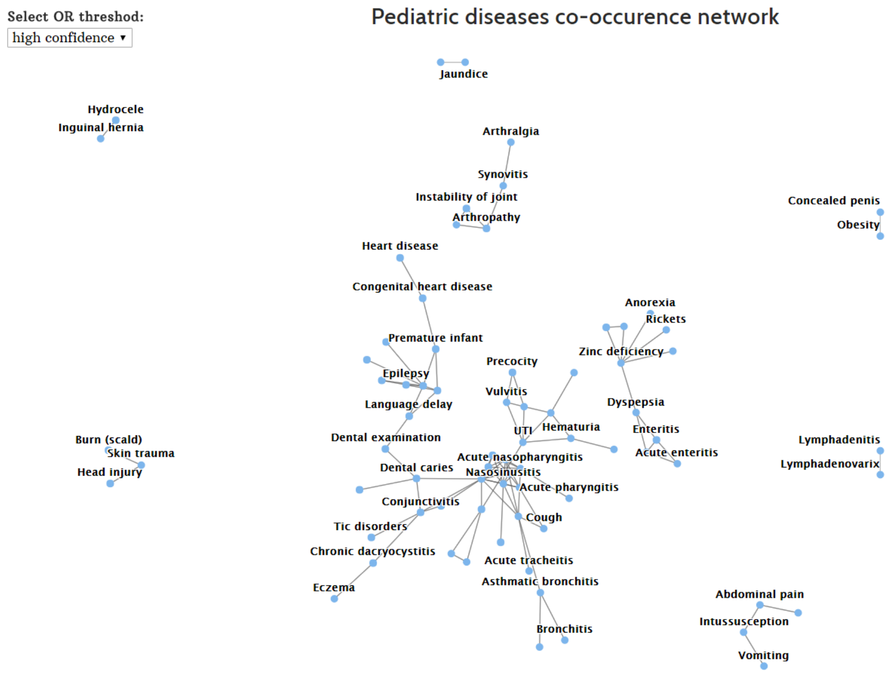

4. Prediction Model

There are two sections (left and right) in the prediction tool page. The right section is a chart that shows the incidence Z-values of the most common pediatric diseases under current customized climate information.

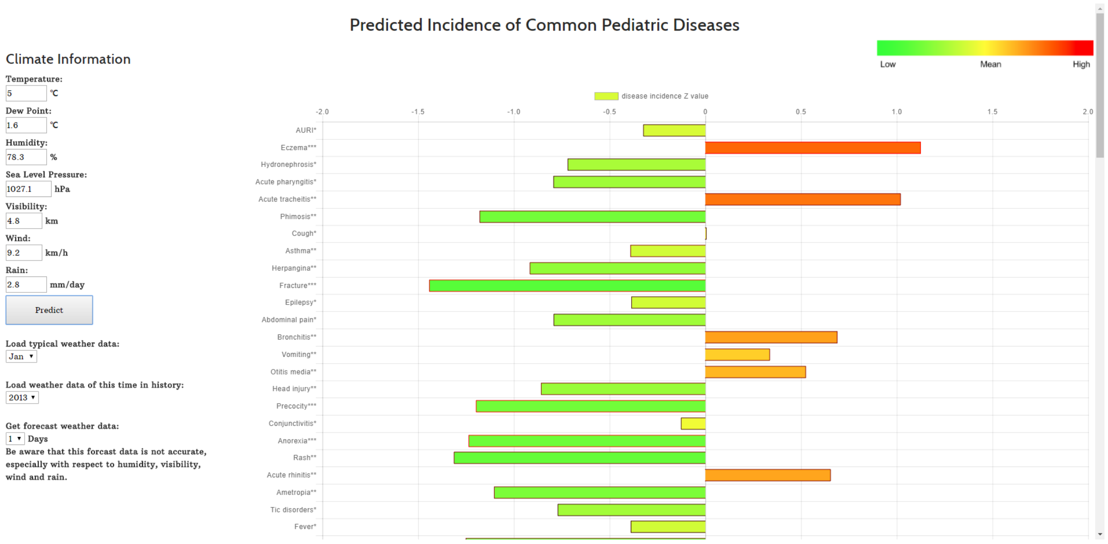

The left section is a form that accepts different types of climate information, such as temperature and dew point. Clicking the predict button will refresh the chart on the right. We also provide some historical climate data and an online weather forecast web service to help users complete the climate information quickly. The goodness of fit of the regression model with the real incidence was classified into 3 levels. The model with a high confidence level is designated by "***" following the disease name. The model with a low confidence level only has "*" after the disease name. Clicking the bar in the chart will open the disease page described below.

5. Disease Page

Both PedMap and the prediction tool can redirect to the disease page when users click the disease bubble or bar. The disease page shows detailed information about this disease, such as the average incidence per month and standard deviation. The percentage of this disease in all pediatric outpatients was also displayed. The incidence Z-value curves and corresponding weather feature curves were also plotted. The age distribution was also shown at the bottom. There was also a prediction tool available for users to predict the incidence Z-value.

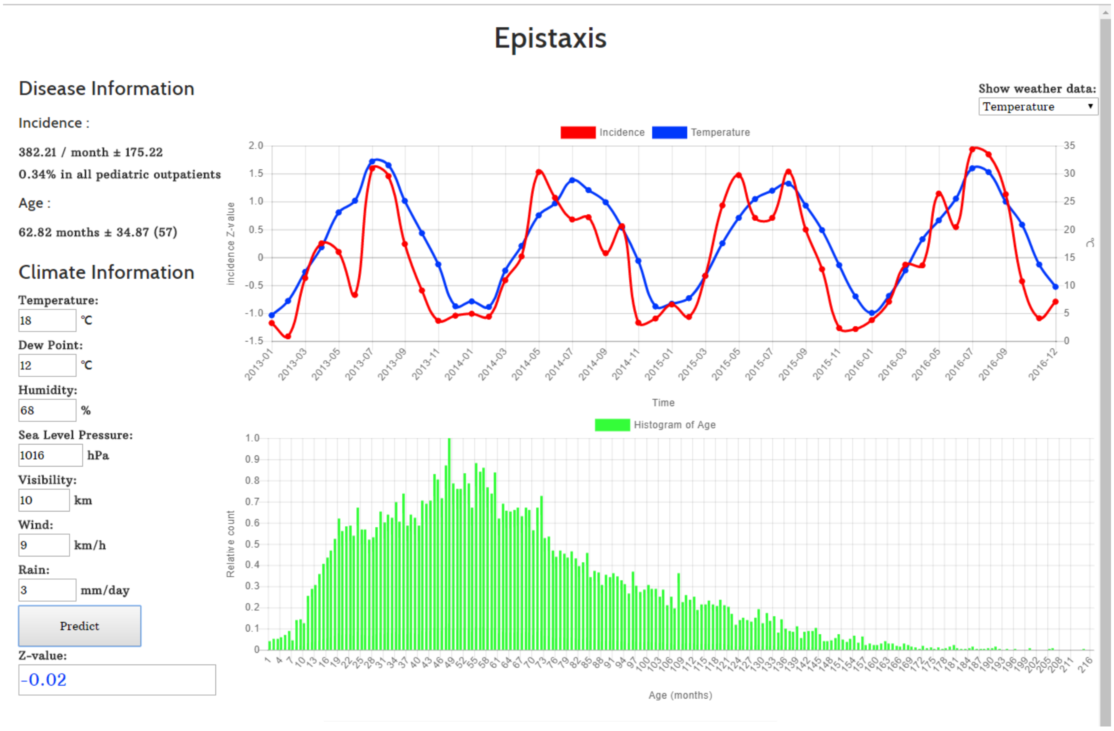

The odds ratio of co-occurrence diseases also will be shown at the bottom of this page. The Y axis show the log2 of odds ratio and the X axis show the log2 of incidence per month. The colors of each diseases are depended on the odd ratios. It also provides two bias adjust dash line which using the most common AURI as a reference.

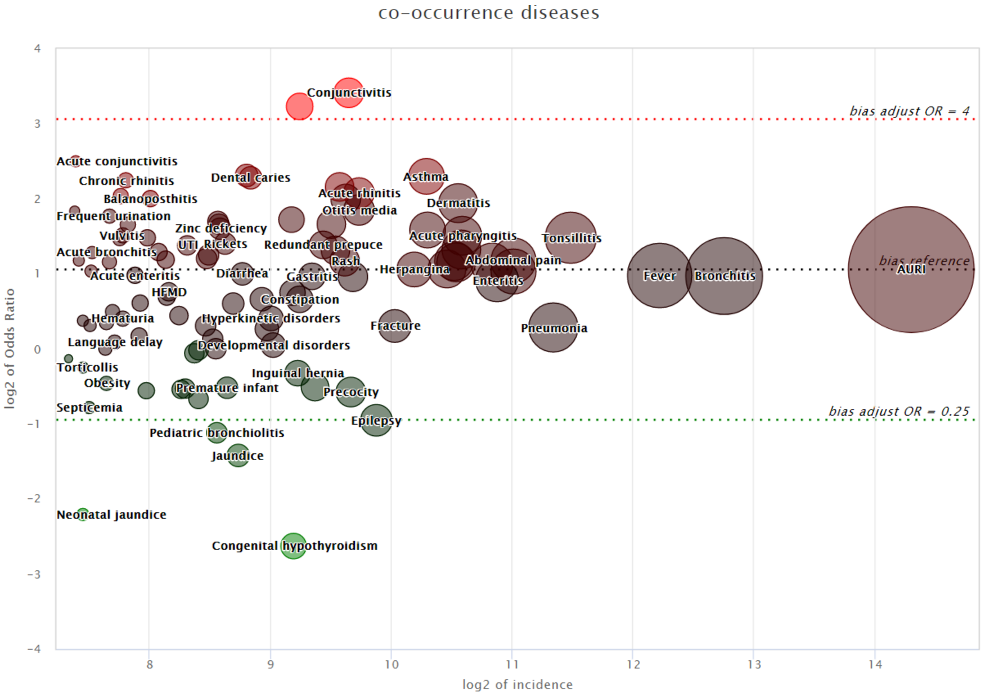

Supplement: Supplementary file 1 — Supplemental Documents [file 41598_2019_54439_MOESM1_ESM.pdf]
